# An EED/PRC2‐H19 Loop Regulates Cerebellar Development

**DOI:** 10.1002/advs.202403591

**Published:** 2024-11-05

**Authors:** Pei‐Pei Liu, Xiao Han, Xiao Li, Shang‐Kun Dai, Ya‐Jie Xu, Lin‐Fei Jiao, Hong‐Zhen Du, Li‐Hua Zhao, Rong‐Feng Li, Zhao‐Qian Teng, Yun‐Gui Yang, Chang‐Mei Liu

**Affiliations:** ^1^ Key Laboratory of Organ Regeneration and Reconstruction State Key Laboratory of Stem Cell and Reproductive Biology Institute of Zoology Chinese Academy of Sciences Beijing 100101 China; ^2^ University of Chinese Academy of Sciences Beijing 100049 China; ^3^ Institute for Stem Cell and Regeneration Chinese Academy of Sciences Beijing 100101 China; ^4^ Key Laboratory of Genomic and Precision Medicine Collaborative Innovation Center of Genetics and Development College of Future Technology Beijing Institute of Genomics Chinese Academy of Sciences Beijing 100101 China; ^5^ Sino‐Danish College University of Chinese Academy of Sciences Beijing 100049 China; ^6^ Jiangsu Key Laboratory of Xenotransplantation Nanjing Medical University Nanjing 211166 China; ^7^ Key Laboratory of Targeted Intervention of Cardiovascular Disease Collaborative Innovation Center for Cardiovascular Disease Translational Medicine Nanjing Medical University Nanjing 211166 China; ^8^ China National Center for Bioinformation Beijing 100101 China

**Keywords:** cerebellum, EED, H19, Motor movement, PRC2

## Abstract

EED (embryonic ectoderm development) is a core subunit of the polycomb repressive complex 2 (PRC2), which senses the trimethylation of histone H3 lysine 27 (H3K27). However, its biological function in cerebellar development remains unknown. Here, we show that EED deletion from neural stem cells (NSCs) or cerebellar granule cell progenitors (GCPs) leads to reduced GCPs proliferation, cell death, cerebellar hypoplasia, and motor deficits in mice. Joint profiling of transcripts and ChIP‐seq analysis in cerebellar granule cells reveals that EED regulates bunches of genes involved in cerebellar development. EED ablation exhibits overactivation of a developmental repressor long non‐coding RNA H19. Importantly, an obvious H3K27ac enrichment is found at *Ctcf*, a trans‐activator of H19, and H3K27me3 enrichment at the H19 imprinting control region (ICR), suggesting that EED regulates H19 in an H3K27me3‐dependent manner. Intriguingly, H19 deletion reduces EED expression and the reprogramming of EED‐mediated H3K27me3 profiles, resulting in increased proliferation, differentiation, and decreased apoptosis of GCPs. Finally, molecular and genetic evidence provides that increased H19 expression is responsible for cerebellar hypoplasia and motor defects in EED mutant mice. Thus, this study demonstrates that EED, H19 forms a negative feedback loop, which plays a crucial role in cerebellar morphogenesis and controls cerebellar development.

## Graphical abstract

The direct evidence is provided that loss of EED/PRC2 impairs cerebellar granule cell proliferation, differentiation, and survival, resulting in a hypoplastic cerebellum in mice. A novel molecular mechanism of PRC2/EED is identified that interacts with autosomal imprinted loci H19, forming a loop to regulate cerebellar morphogenesis and control cerebellar development.

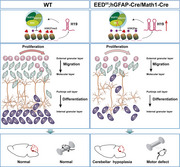

## 1 Introduction

The cerebellum plays key roles in motor control and learning sensorimotor tasks. Developmental deficits of the cerebellum usually lead to motor and higher cognitive disorders, including impaired balance control, spatial memory, sensory/motor learning, and speech.^[^ ^1^ ^]^ Recent studies have suggested diverse epigenetic regulators in the development of the mammalian cerebellum and cerebellar‐dependent behaviors.^[^ ^2^ ^]^ Notably, mutations of epigenetic regulators often cause a wide range of neurodevelopmental disorders, ranging from autism and intellectual disability to epilepsy, generating wide interest in understanding how these key epigenetic regulators govern the development and function of the brain including in the cerebellum.^[^ ^3^ ^]^

Polycomb repressive complex2 (PRC2) has emerged as a transcriptional repressor that epigenetically modifies chromatin and participates in the determination and maintenance of cell fate during brain development.^[^ ^4^ ^]^ PRC2 typically contains the core components EZH2 (enhancer of zeste homolog 2), EED (embryonic ectoderm development), and SUZ12 (suppressor of zeste 12) to catalyze histone H3 lysine 27 trimethylation (H3K27me3).^[^ ^5^ ^]^ Deletion of *Ezh2* results in H3K27me3 and profound transcriptional dysregulation in cerebellar precursors, including dysregulation of a series of transcription factors that are involved in cerebellar neuronal differentiation.^[^ ^6^ ^]^ As a result, *Ezh2* knockout mice exhibit severe cerebellar hypoplasia.^[^ ^6^ ^]^ Consistently, the loss of PRC2 in postnatal cerebellar Purkinje cells (PCs) results in cerebellar degeneration by the upregulation of genes that are critical for PCs function and survival.^[^ ^7^ ^]^ EED mediates the process of gene silencing and chromatin condensation through binding to H3K27me3,^[^ ^8^ ^]^ and depletion of EED causes a global increase in histone H3 and H4 acetylation.^[^ ^9^ ^]^ Our previous research has shown that loss of EED in the embryonic neural stem cells (NSCs) leads to postnatal lethality, impaired migration of granule cells, and malformation of the dentate gyrus through targeting SOX11.^[^ ^9^ ^]^ Meanwhile, we also observed that mice with EED knockout in embryonic NSCs displayed severe malformation of the cerebrum,^[^ ^10^ ^]^ however, whether and how EED regulates cerebellar development remains to be determined.

In this study, we for the first time provide evidence showing that genetic ablation of EED in either NSCs or cerebellar granule cell progenitors(GCPs) disrupted cerebellar morphogenesis and led to severe motor impairments in mice. We then identified that EED‐mediated long non‐coding RNA H19 was essential for the proliferation, migration, and differentiation of GCPs and proper motor behavior. Moreover, we highlighted that H19 regulates the level of H3K27me3 and EED expression. Our data indicate that EED and H19 form a loop during the epigenetic regulation of cerebellum development. Additionally, this provides a better understanding of EED mutation‐associated human diseases.

## 2 Results

### 2.1 Loss of EED in the Cerebellum Results in Cerebellar Hypoplasia and Severe Motor Impairments

**Figure 1:**
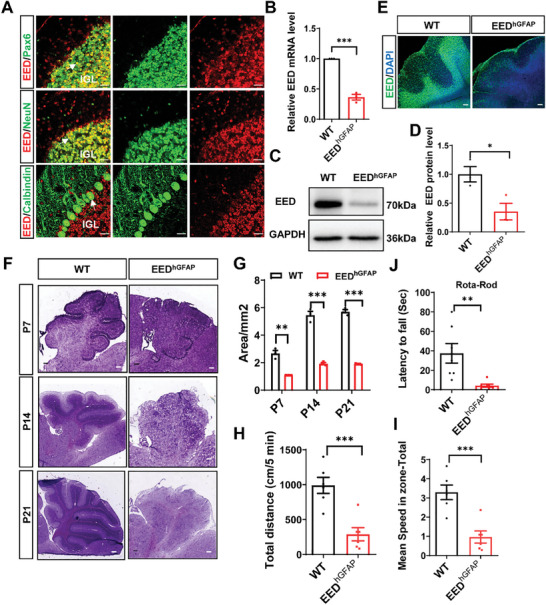
*Eed* cKO mice develop motor impairments. A) Top, co‐immunostaining of EED (red) with Pax6 (green) in the cerebella at P21. Middle, co‐immunostaining of EED (red) with NeuN (green) in the IGL of cerebella at P21. Bottom, cerebellar sections from P21 mice were stained with antibodies against EED (red) and Calbindin(green). The co‐localization cells are defined by arrowheads. IGL, internal granule cell layer. Scale bars, 20 µm. B)Highly efficient depletion of EED protein from the nervous system in *hGFAP‐Cre* mediated conditional knockout mice as shown by qRT‐PCR using P14 cerebellar lysates. ^***^ *p *< 0.001, *n* = 3, mean ± SEM. C)Western blot analysis of EED protein from the nervous system in *hGFAP‐Cre* mediated conditional knockout mice using P14 cerebellar lysates. D) Quantification of the density of the EED protein bands by normalization to the intensity of GAPDH bands in C. ^*^ *p *< 0.05, *n* = 4, mean ± SEM. E) Immunostaining of EED (green) in P21 cerebella of WT and EED^hGFAP^ mice. DNA was stained with DAPI. Scale bars, 50 µm. F) Violet staining of cerebellar sections from WT and EED^hGFAP^ mice shows dysmorphology in mutant mice during P7‐P21. Scale bars, 500 µm. G) Area of sagittal sections of the cerebellar vermis in WT and EED^hGFAP^ mice at the indicated postnatal developmental stages. ^**^ *p *< 0.01, ^***^ *p *< 0.001, *n* = 3, mean ± SEM. (H) EED^hGFAP^ mice had reduced locomotivity to WT littermate mice in an open field test over a 5 min period. ^***^ *p *< 0.001, *n* = 6, mean ± SEM. I) EED^hGFAP^ mice had reduced mean speed to WT littermate mice in an open field test over a 5 min period. ^***^ *p* < 0.001, *n* = 6, mean ± SEM. J)The mean latency of mice to fall from the rotarod. Note the significant difference between the EED^hGFAP^ and WT mice. ^**^ *p *< 0.01, *n* = 7, mean ± SEM.

To investigate the contribution of EED to cerebellar development, we first performed immunostaining assays to analyze the co‐expression of EED with cell type‐specific markers in the cerebellum of wild‐type mice. We observed that the EED expression level was relatively higher in the cerebellum in the early postnatal stage, and gradually decreased thereafter (Figure S1A–C, Supporting Information). EED was still highly expressed in cerebellar granule progenitor cells (Pax6^+^), cerebellar granule cells (NeuN^+^), and Purkinje cells (Calbindin^+^) in P21 cerebellum (**Figure** **1**A), indicating that EED could play an important role in cerebellar development.

Next, we utilized a well‐characterized human *hGFAP‐Cre* transgenic mouse line (*hGFAP‐Cre*), which is specifically detected in radial glial cells from embryonic day 12.5 (E12.5), to generate EED conditional knockout mice (*hGFAP‐Cre*;*Eed^f/f^*; EED^hGFAP^; *Eed* cKO) (Figure S1D, Supporting Information). Real‐time qPCR and western blot analysis of whole cerebellum lysates showed an efficient reduction of EED in the EED^hGFAP^ mice (Figure 1B–D). Immunostaining assays confirmed ablation of EED in granule cells of EED^hGFAP^ mice (Figure 1E). Consistent with our previous findings, EED^hGFAP^ mice displayed reduced hippocampus size, which was apparent at postnatal day 7(P7) and more evident at P14 and P21.^[^ ^5^ ^]^ Crystal violet staining of cerebellar sections confirmed that EED^hGFAP^ mice severely diminished cerebellar volume with the most striking hypoplasia presenting in lobules, compared with control littermates at P14 and P21(Figure 1F,G).

To validate the phenotype of EED deletion in cerebellar development, we decided to use *Math1*‐Cre to specifically delete *Eed* in GCPs (Figure S1D, Supporting Information). Quantiative real‐time PCR (qRT‐PCR) and immunoblotting assays verified the successful ablation of EED in (*Math1‐Cre; Eed^f/f^*, hereafter called EED^Math1^) homozygous mutant mice (Figure S1E–G, Supporting Information). Immunostaining assays confirmed ablation of EED in granule cells of EED^Math1^ mice (Figure S1H, Supporting Information). Consistent with EED^hGFAP^ mice, we found notable cerebellar foliation defects in EED^Math1^ mice compared with control mice from P7 to P21 (Figure S1I,J, Supporting Information).

Next, we asked whether the cerebellar defects upon the loss of EED were sufficient to cause behavioral abnormalities in mice. Compared with control mice, EED^hGFAP^ mice showed reduced total distance traveled (Figure 1H) and decreased mean speed (Figure 1I) in the open field fest. Moreover, EED^hGFAP^ mice performed significantly worse than control mice on a revolving rotarod (Figure 1J). As in the EED^hGFAP^ mice, the EED^Math1^ mutants also showed impairment of motor performance (Figure S1K–M, Supporting Information). These results support that loss of EED in the cerebellum causes cerebellar hypoplasia and severe motor impairments.

### 2.2 EED Deletion Impairs GCPs Proliferation and Induces Apoptosis of Newborn Granule Cells

**Figure 2:**
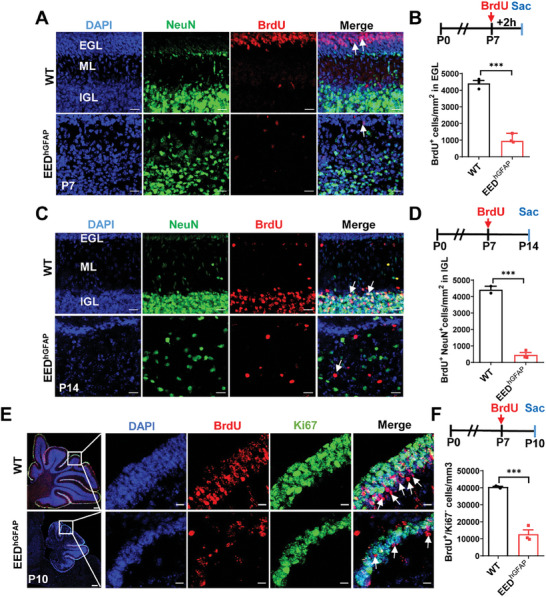
EED ablation exhibits defective GCP proliferation and leads to cell death in vivo. A) GCPs proliferation in EGL was labeled with anti‐BrdU in WT and EED^hGFAP^ mice at P7. Differentiated granule cells were co‐immunolabeled with anti‐NeuN antibodies. Arrowheads show BrdU‐positive cells. Scale bars, 20 µm. EGL, external granule cell layer. IGL, internal granule cell layer. ML, molecular layer. B) GCPs proliferation in EGL was evaluated after a 2 h chase following BrdU injection in WT and EED^hGFAP^ mice at P7. Quantification of BrdU‐positive cells within the EGL. ^***^ *p* < 0.001, *n* = 3, mean ± SEM. C) Migrating GCPs in WT and EED^hGFAP^ mice were labeled with anti‐BrdU. Differentiated granule cells were co‐immunolabeled with anti‐NeuN antibodies. Arrowheads show a decreased number of BrdU‐positive and NeuN^+^ cells in the IGL, indicating impaired migration. Scale bar, 20 µm. D) Tracing of granule neuron migration was evaluated in WT and EED^hGFAP^ mice that were injected with BrdU at P7 and chased until P14. Quantification of BrdU‐positive and NeuN^+^ cells in the IGL. ^**^ *p* < 0.01, *n* = 3, mean ± SEM. E) BrdU (red), Ki67 (green), and DAPI (blue) immunofluorescent staining of WT and EED^hGFAP^ mice cerebellums at P10. White frames indicate folium at P10. Higher magnification of folium is shown in the adjacent right panels. Arrowheads indicate BrdU^+^Ki67^−^ cells. Left, Scale bars, 200 µm. Right, Scale bars, 10 µm. F) Apoptosis of newborn granule cells was evaluated in WT and EED^hGFAP^ mice that were injected with BrdU at P7 and chased 72 h after BrdU administration at P10. Proportion of BrdU^+^/Ki67^−^ cells in the EGL of WT and EED^hGFAP^ mice 72 h post–BrdU injection. ^***^ *p* < 0.001, *n* = 3, mean ± SEM.

To assess the proliferation potential of GCPs with EED mutant, we labeled dividing neurons with 5‐bromo‐2′‐deoxyuridine (BrdU) at P7, a peak stage for postnatal GCP proliferation, and monitored BrdU incorporation in the external granule cell layer (EGL) after a 2 h chase. At P7, the EED^hGFAP^ EGL exhibited a marked decrease in the number of BrdU‐incorporating cells compared with the control (**Figure** **2**A,B). Meanwhile, NeuN and nuclear staining results demonstrated the severe disorganization of the granule cell layer and the increased number of cells in the ML of EED^hGFAP^ mice (Figure 2A).

We then analyzed the effect of EED ablation on the migration of GCPs in EGL. We labeled migrating neurons with BrdU and analyzed the number of BrdU‐incorporated cells in the internal granule cell layer (IGL) after a 7‐day chase (Figure 2C,D). At P14, a significantly reduced number of BrdU^+^NeuN^+^ incorporating cells was detected in the EED mutant IGL (Figure 2D). Moreover, fewer Ki67‐negative cells among BrdU‐positive cells were exhibited in the EGL of EED‐deficient mice with 72 h BrdU labeling at P7, indicating that loss of EED prevented appropriate cell cycle exit of GNPs in vivo. Less differentiated and more apoptotic granule cells were then observed in EED mutant mice, again supporting the failure of cell cycle exit of EED mutant GNPs (Figure 2E,F). Indeed, TUNEL staining showed elevated levels of apoptosis in EED^hGFAP^ mice compared with controls at P7 (Figure S2A,B, Supporting Information) and P14 (Figure S2C,D, Supporting Information), especially in the EGL region. Our data indicate that cerebellar hypoplasia in EED mutant mice may come from a failure of GCP differentiation and an enhancement of cell death.

In line with the above observations, we found that the proliferation of GCPs was decreased in EED^Math1^ EGL by the BrdU assay (Figure S3A,B, Supporting Information). We also detected a significantly decreased number of BrdU^+^ NeuN^+^cells in the IGL in EED^Math1^ mutants compared with the controls, suggesting impaired self‐renewal and migration of GCPs with the loss of EED (Figure S3C,D, Supporting Information). In addition, the cerebella of EED^Math1^ mice also displayed increased levels of apoptotic cell death at P7 and P14, as evaluated by TUNEL staining (Figure S3E–G, Supporting Information). Together, these data suggest that the alteration in the folia layering and structure in EED mutant mice may be contributed by the impairments of proliferation, migration, and cell death of GCPs.

### 2.3 EED Regulates a Transcriptional Network Essential for Cerebellum Development

**Figure 3:**
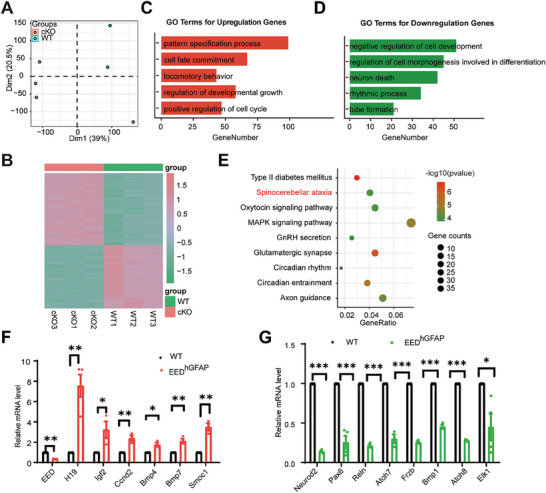
Up‐regulation of H19 in EED^hGFAP^ cerebellum. A) PCA cluster analysis of WT (green) and *Eed* cKO(red) mice cerebellum RNA‐seq samples. B) Heatmap of differentially expressed genes in WT and *Eed* cKO mice cerebellum samples. C) GO biological process analysis of upregulated genes (cKO vs WT). D) GO biological process analysis of downregulated genes (cKO versus WT). E) KEGG analysis of genes with down‐regulated expression in P14 *Eed* cKO mice cerebellums as compared with WT mice. F) qRT‐PCR analyses of genes that are up‐regulated expressed in *Eed* cKO cerebellar cells at P14. ^*^ *p* < 0.05, ^**^ *p *< 0.01, *n* ≥ 3, mean ± SEM. G) qRT‐PCR analyses of representative cerebellum development genes that are down‐regulated expressed in *Eed* cKO cerebellar cells at P14. ^*^ *p* < 0.05, ^***^ *p *< 0.001, *n* ≥ 3, mean ± SEM.

To investigate the molecular mechanisms underlying the cerebellar developmental defects in EED mutant mice, we next performed RNA sequencing‐based transcriptome analysis using cerebellar tissues isolated from WT and *Eed* cKO mice at P14. Using principal component analysis (PCA) and Pearson correlation coefficient, we found the inherent similarity in biological replicates and clear separation of gene expression between WT and *Eed* cKO mice (**Figure** **3**A; Figure S4, Supporting Information). Using DESeq2, we identified a group of 3149 differentially expressed genes (abs (fold change) >1.5, *p* < 0.05), including 1722 upregulated and 1427 downregulated genes in *Eed* cKO mice as compared with the controls (Figure 3B). Functional classification of differentially expressed genes (DEGs) by Gene Ontology （GO） revealed that upregulated genes were enriched in the pattern specification process, cell fate commitment, locomotory behavior, regulation of developmental growth, and positive regulation of cell cycle (Figure 3C). In contrast, downregulated genes were involved in processes related to negative regulation of cell development, regulation of cell morphogenesis involved in differentiation, neuron death, rhythmic process, and tube formation (Figure 3D).

Interestingly, Kyoto Encyclopedia of Genes and Genomes (KEGG) analysis of genes downregulated upon loss of EED revealed neuronal functions and spinocerebellar ataxias are the most enriched biological processes (Figure 3E). Gene Set Enrichment Analysis (GSEA) revealed that the differentially expressed genes in *Eed*‐deficient cerebellar cells were enriched in gene signatures associated with cell fate specification, central nervous system neuron differentiation, and morphogenesis (Figure S4B–D, Supporting Information). qRT‐PCR analysis confirmed these significantly up‐regulated genes are a large set of genes specifically expressed in neural development, such as *H19*, *Igf2*, *Ccnd2*, *Bmp4*, *Bmp7*, *Smoc2* (Figure 3F). In contrast, qRT‐PCR analysis of the down‐regulated genes with EED deficiency in *Eed* cKO cerebellums, including essential factors for cerebellar development such as *Neurod2, Pax6, Atoh7*, *Reln*, *Frzb*, *Bmp1*, *Atoh8, Elk1* (Figure 3G). Taken together, these data indicated that EED is required for cerebellum development.

### 2.4 EED Interacts with H19 to Regulate Cerebellum Development

We identified the long non‐coding RNA H19 was the most significantly upregulated gene among the differentially expressed genes, whose expression level was approximately eight‐fold higher in the *Eed* cKO mouse cerebellum than in wild‐type mouse cerebellum at P14 (Figure 3F). H19 is a maternally expressed imprinted gene that regulates fetal development and has an integral role in the development of many tissues, including the brain.^[^ ^11^ ^]^ Studies have shown that the imprinted gene cluster Igf2/H19 is an evolutionarily conserved domain and is essential for mammalian development.^[^ ^12^ ^]^ The mouse *Igf2* (insulin‐like growth factor 2) and H19 genes are located near each other on chromosome 7 and are reciprocally imprinted.^[^ ^13^ ^]^ Indeed, we found that *Igf2* was also upregulated in the *Eed* cKO mice cerebellum (Figure 3F). Importantly, in humans, excessive expression of H19 is linked to Silver–Russell dwarfism, a development retardation disorder characterized by intrauterine growth restriction.^[^ ^11^, ^14^ ^]^ All these results suggest that overexpression of H19 may contribute to the developmental defects observed in *Eed* cKO mice.

**Figure 4:**
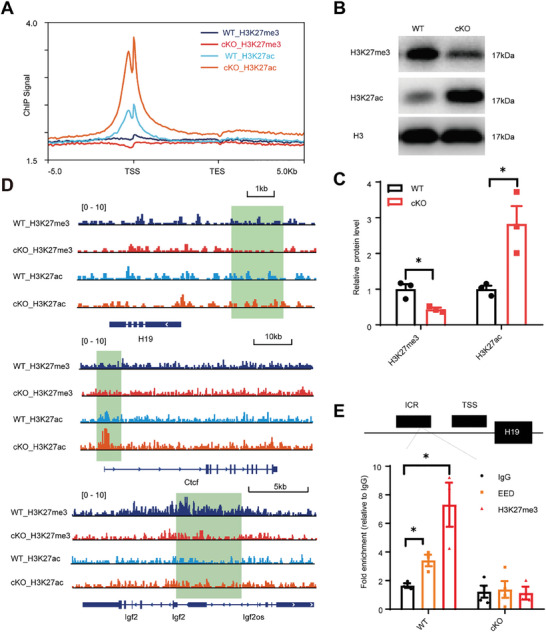
EED controls the transcriptional program necessary for cerebellum development. A) Average genome‐wide occupancies of H3K27ac and H3K27me3 within ±5 kb of the gene body of all GENCODE. B) The levels of H3K27me3 and H3K27ac in isolated cerebellar tissues from P14 WT and EED^hGFAP^ mice were measured by immunoblotting. C) Quantified the density of the histone protein bands by normalization to the density of total histone H3. ^*^ *p* < 0.05, *n* = 3, mean ± SEM. D) ChIP‐seq binding profiles of H3K27me3 and H3K27ac at H19 ICR (loci −2–4 kb), *Ctcf*, *Igf2* as indicated in WT and EED cKO cerebellum at P14. The regions marked in green show the gain or loss of H3K27me3 and H3K27ac. E) ChIP‐qPCR assessment of EED and H3K27me3 enrichment at ICR regions of H19. *n* = 3–4 wells per condition. ^*^ *p* < 0.05, mean ± SEM.

To dissect the relationship between the differentially expressed genes and the occupancy of H3K27me3, H3K27ac, and EED, we performed chromatin–immunoprecipitation coupled to sequencing (ChIP‐seq) using freshly isolated P14 cerebellar tissues. We found that the enrichment of H3K27me3 was decreased and H3K27ac was highly increased in *Eed* cKO mice (Figure S5A,B, Supporting Information). Meta‐analysis of ChIP‐seq data showed that both *Eed* cKO and WT displayed enrichment of H3K27me3 and H3K27ac in promoters (±5 kb from the transcription start site [TSS]), and there was a reduction in H3K27me3 and an obvious increased in H3K27ac at TSS regions (**Figure** **4**A; Figure S5A,B, Supporting Information). Consistent with this, western blot analyses of extracts prepared from mouse cerebellar tissues showed a global loss of H3K27me3 and a markedly increased H3K27ac (Figure 4B,C). These data suggest that EED inactivation results in the loss of H3K27me3 and the gain of H3K27ac.

Interestingly, we found that H3K27me3 enrichment decreased along the imprinting control region (ICR) of H19, located 2–4 kb upstream of the H19 gene upon EED knockout (Figure 4D). To confirm this, we calculated the change in H3K27me3 density within the 4 kb region upstream of the H19 transcription start site (TSS) in both WT and cKO groups. Our results demonstrated a clear pattern of reduced H3K27me3 density upstream of H19 genes in *Eed* cKO mice (Figure S5C, Supporting Information). To further investigate whether H19 expression is regulated by H3K27me3, we performed chromatin immunoprecipitation (ChIP) followed by quantitative real‐time PCR. Our observations revealed that the ICR of H19 is occupied by less H3K27me3 in the absence of EED. Furthermore, the activating histone mark H3K27ac increased at the promoter of *Ctcf*, a trans‐activator of H19, in EED cKO mice compared with WT mice (Figure 4D). In addition, EED inactivation decreased the H3K27me3 signal at the promoter of *Igf2* (Figure 4D). Next, we probed whether H19 expression is directly regulated by EED using ChIP‐qPCR. We observed an enrichment of EED at the ICR which is located upstream of H19 in the WT mice cerebellum, but less enrichment was observed in *Eed* cKO mice (Figure 4E), indicating that EED is directly bound to the ICR of H19. These data suggest that EED binds to H19 dependent on H3K27me3.

### 2.5 H19 and EED Form a Loop to Repress the Proliferation and Differentiation of GCPs

**Figure 5:**
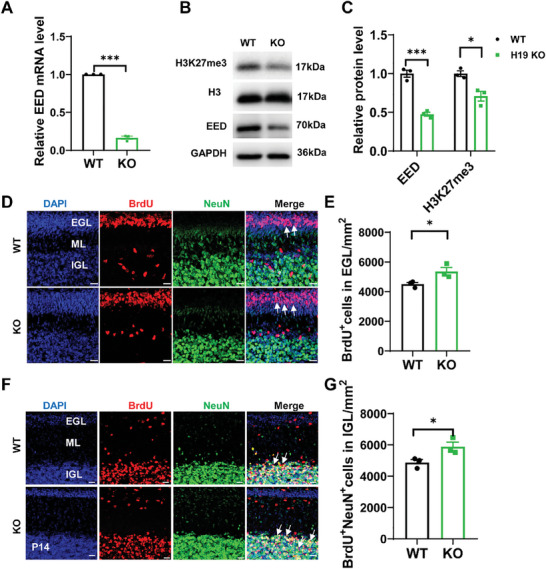
H19 deletion promotes the proliferation and migration of GCPs. A) Highly reduced EED protein from H19 KO mice as shown by qRT‐PCR using P14 cerebellar lysates. ^***^ *p *< 0.001, *n* = 3, mean ± SEM. B) Western blot analysis of EED protein and H3K27me3 from H19 KO mice using P14 cerebellar lysates. C) Quantification of the density of the EED protein bands by normalization to the intensity of GAPDH bands in B. Quantification of the density of the H3K27me3 protein bands by normalization to the intensity of H3 bands in B. ^*^ *p *< 0.05, ^***^ *p *< 0.001, *n* = 3, mean ± SEM. D) GCPs proliferation in EGL was labeled with anti‐BrdU in WT and H19 KO mice after a 2 h chase following BrdU injection at P7. Differentiated granule cells were co‐immunolabeled with anti‐NeuN antibodies. Arrowheads show BrdU‐positive cells. Scale bars, 20 µm. EGL, external granule layer. E) Quantification of BrdU‐positive cells within the EGL. ^*^ *p* < 0.05, *n* = 3, mean ± SEM. F) Migrating GCPs in WT and H19 KO mice were labeled with anti‐BrdU. Differentiated granule cells were co‐immunolabeled with anti‐NeuN antibodies. Tracing of granule neuron migration was evaluated in WT and H19 KO mice that were injected with BrdU at P7 and chased until P14. Arrowheads show BrdU‐positive and NeuN^+^ cells in the IGL. Scale bars, 20 µm. G) Quantification of BrdU^+^NeuN^+^ cells in the IGL. ^*^ *p* < 0.05, *n* = 3, mean ± SEM. IGL, internal granule cell layer.

To determine the contribution of H19 to cerebellar development, we first tested expression levels of H19 during development and found relatively a higher level of the H19 transcript in the P0 WT cerebellum than in the P21 WT cerebellum (Figure S6A, Supporting Information). To explore whether EED regulates cerebellum foliation patterning via H19, we generated H19 mutant mice by CRISPR/Cas9 gene editing system. Because mice that lack H19 were observed to be homozygous viable, we used H19 homozygous (H19 KO) for further analysis. We observed efficient elimination of H19 in H19 KO mice by qRT‐PCR (Figure S6B, Supporting Information). Previous research indicates that H19 has been shown to regulate EZH2 expression depending on H3K27me3 in cancer cells.^[^ ^15^ ^]^ We speculate whether it is possible for H19 to regulate other components of PRC2, such as the EED. To test this hypothesis, we tested EED and H3K27me3 levels in H19 KO mice. Consistent with our speculations, our results indeed demonstrated that the expression of EED and the level of H3K27me3 were reduced in the cerebella of H19 homozygous mice (**Figure** **5**A–C). In addition, ChIP data confirmed that H19 was enriched in the immunoprecipitation of endogenous EED (Figure 4E). These results indicate that H19 regulates EED‐mediated histone modification in the cerebellum development.

Because the cerebellar phenotype of H19 knockout mice has not been reported so far, we first explored the functions of H19 in cerebellar GCPs. We found that deletion of H19 resulted in promoting cell proliferation effects as the number of BrdU‐incorporating cells within the EGL was increased, as shown in Figure 5D,E. We then investigated the effect of H19 ablation on the migration of GCPs in the EGL. We labeled migrating neurons with BrdU at P7 and analyzed the number of BrdU^+^NeuN^+^ cells in the IGL at P14. These H19 ablation mice displayed an increased number of BrdU‐positive cells in the IGL, indicating abnormal GNP migration in mutants (Figure 5F,G). In addition, the cerebella of H19 ablation mice displayed decreased levels of apoptotic cell death at P7 while mostly unaltered at P14, as evaluated by TUNEL staining (Figure S6C–E, Supporting Information). These data indicate that EED and H19 form a loop, which balances the proliferation and differentiation and apoptosis of GCPs.

### 2.6 H19 Downregulation Partially Rescues Motor Defects Caused by *Eed* Loss

**Figure 6:**
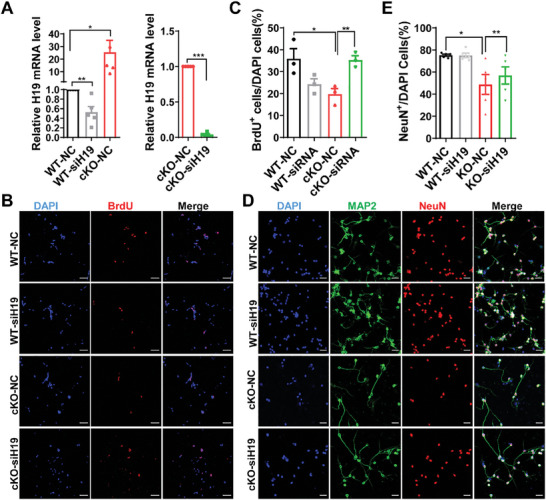
H19 downregulation rescues the proliferation defect and cell survival in *Eed* cKO GCPs and cerebellar granule cells. A) RT‐qPCR analyses of H19 in GCP cells at 48 h after transfection with NC or siRNA‐H19 (*n* = 3 experiments). GCP cells were isolated from WT and *Eed* cKO mice at P6. ^*^ *p* < 0.05, ^**^ *p *< 0.01, ^***^ *p *< 0.001, mean ± SEM. B) Immunostaining for BrdU(red) in WT and *Eed* cKO GCPs transfection with NC or siRNA‐H19 for 48 h (*n* > 3 experiments). Scale bars, 50 µm. C) Quantification of BrdU^+^ cells (right) in WT and *Eed* cKO GCPs transfection with NC or siRNA‐H19 for 48 h (*n* > 3 experiments). ^*^ *p *< 0.05, ^**^ *p *< 0.01, mean ± SEM. D) Immunostaining for MAP2(green) and NeuN(red) in WT and *Eed* cKO GCPs transfection with NC or siRNA‐H19 for 48 h (*n* > 3 experiments). Scale bars, 20 µm. E) Quantification of NeuN^+^ cells (right) in WT and *Eed* cKO GCPs transfection with NC or siRNA‐H19 for 48 h (*n* > 3 experiments). ^*^ *p* < 0.05, ^**^ *p *< 0.01, mean ± SEM.

To further determine the role of the H19 upregulation in *Eed* cKO mice involved in the cerebellum defects, we isolated GCPs in vitro to investigate their impact on the foliation pattern by exploring their proliferation potential. We found that the expression of H19 was significantly upregulated in *Eed* cKO GCPs, compared with that in GCPs isolated from WT mice (**Figure** **6**A). Transfection of GCPs with siRNA‐H19 led to robust reduction of endogenous H19 transcripts (Figure 6A) and knockdown of H19 in *Eed* cKO GCPs dramatically improved their proliferation potentials compared with *Eed* cKO GCPs transfected with scramble RNA (NC) (Figure 6B,C). Then, we suppressed H19 using siRNAs in each set of cerebellum granule neurons (CGNs) to evaluate the effects on cell survival. We found that H19 siRNA treatment improved CGNs survival relative to a scrambled siRNA control in *Eed* cKO CGNs (Figure 6D,E). Consistently, we found that H19 siRNA treatment improved CGNs survival relative to a scrambled siRNA control in *Eed* cKO CGNs by TUNEL staining (Figure S7A,B, Supporting Information).

**Figure 7:**
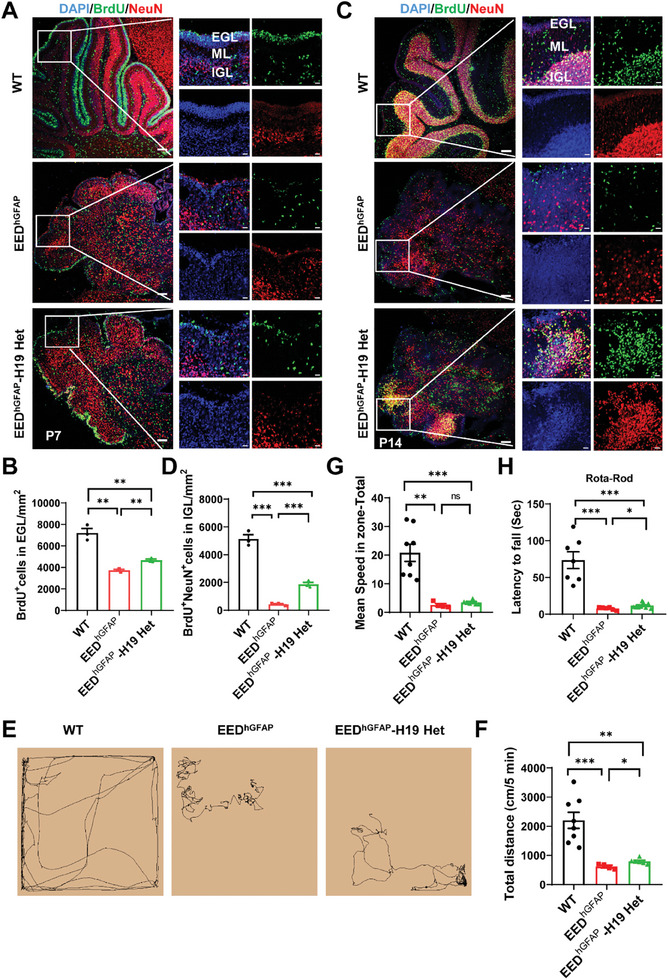
H19 downregulation partially rescues the motor defect in *Eed* cKO mice. A) Immunostaining for BrdU(green), NeuN(red), and DAPI in WT, EED^hGFAP^, and EED^hGFAP^‐H19 Het mouse cerebellums 2 h after BrdU injection at P7. White frames indicate folium at P7. Higher magnification of folium is shown in the adjacent right panels. Left: Scale bars, 100 µm. Right, Scale bars, 20 µm. B) Quantification of BrdU^+^ cells in EGL in WT, EED^hGFAP^, and EED^hGFAP^‐H19 Het mouse cerebellums. ^**^ *p *< 0.01, mean ± SEM. C) Migrating GCPs in WT, EED^hGFAP^, and EED^hGFAP^‐H19 Het mouse cerebellums were labeled with anti‐BrdU. Differentiated granule cells were co‐immunolabeled with anti‐NeuN antibodies. Tracing of granule neuron migration was evaluated in WT, EED^hGFAP^, and EED^hGFAP^‐H19 Het mouse cerebellums that were injected with BrdU at P7 and chased until P14. White frames indicate folium at P14. Higher magnification of folium is shown in the adjacent right panels. Left: Scale bars, 100 µm. Right, Scale bars, 20 µm. D) Quantification of BrdU^+^NeuN^+^ cells in the IGL. ^*^ *p* < 0.05, *n* = 3, mean ± SEM. IGL, internal granule cell layer. E) Representative motion trail from the open field test. F) EED^hGFAP^‐H19 Het mice had significant improvement in locomotivity to EED^hGFAP^ littermate mice in an open field test over a 5 min period. ^*^ *p *< 0.05, ^**^ *p *< 0.01, ^***^ *p *< 0.001, *n* = 4–6, mean ± SEM. G) EED^hGFAP^‐H19 Het mice had no significant mean speed to EED^hGFAP^ littermate mice in an open field test over a 5 min period. ^**^ *p *< 0.01, ^***^ *p *< 0.001, *n* = 4–6, mean ± SEM. H)The mean latency of mice to fall from the rotarod. Note the significant difference between the EED^hGFAP^ and EED^hGFAP^‐H19 Het mice. ^*^ *p *< 0.05, ^**^ *p *< 0.01, *n* = 6–9, mean ± SEM.

In addition, we generated compound conditional mutant mice missing EED and one allele of H19(EED^hGFAP^‐H19 Het) for further analysis because of the low birth ratio of EED‐H19 double KO mice. Again, we found that EED^hGFAP^‐H19 Het dramatically improved cellular proliferation (**Figure** **7**A,B) and migration (Figure 7C,D) in *Eed* cKO mice. Furthermore, EED^hGFAP^‐H19 Het partially alleviated cell apoptosis in the *Eed* cKO mice (Figure S8A‐D, Supporting Information). Next, we asked whether behavioral abnormalities in mice upon the loss of EED were recovered by H19 repression. Compared with EED^hGFAP^ mice, EED^hGFAP^‐H19 Het showed significant improvement in total distance traveled (Figure 7E–G) in the open field test. In another test, EED^hGFAP^‐H19 Het performed significantly better than EED^hGFAP^ mice on a revolving rotarod (Figure 7H). These data support the idea that excessively active H19 is responsible for developmental defects in the *Eed* cKO cerebellum.

## 3 Discussion

Epigenetic factors have recently been recognized as important regulators in cerebellar development. However, we still know very little about the epigenetic mechanisms underlying cerebellar development. In the present study, we provide strong evidence showing that loss of EED/PRC2 impairs the proliferation, migration, and survival of cerebellar granule cells, resulting in a hypoplastic cerebellum in mice. EED‐mediated repression of long non‐coding RNA H19 is responsible for the deficits of cerebellar development (Figure S9, Supporting Information).

### 3.1 EED/PRC2 is Required for Normal Cerebellar Neurogenesis

PRC2 plays crucial roles in cell growth, neuronal identity, proliferation, migration, differentiation, and maturation.^[^ ^8^, ^16^ ^]^ A previous study has shown that EED/PRC2 is a critical epigenetic programmer of CNS myelination and repair.^[^ ^17^ ^]^ EED is required for the Subventricular Zone (SVZ) neurogenesis, and loss of EED impairs neuronal differentiation and leads to malformation of the dentate gyrus.^[^ ^5^, ^18^ ^]^ Here our study demonstrated that *Eed* loss‐of‐function decreased the proliferation of granule precursor cells of the EGL. Granule precursor cells contribute to the proper cerebellar growth and shape the foliation pattern throughout embryonic and early postnatal stages.^[^ ^19^ ^]^ In both rodents and humans, cerebellar development extends a long period from the early embryonic stage until the first postnatal weeks (in mice) or years (in humans). Loss of granule cells leads to cerebellar hypoplasia, as seen in *Chd7* and *Nf1* mutant mice.^[^ ^20^ ^]^ Consistent with this, due to the decreased proliferation potential of granule cells at postnatal stages, loss of EED in progenitor cells and granule cells ultimately led to a hypoplastic cerebellum in *Eed* cKO mice.

In our study, we found that EED^hGFAP^ mice show more severe cerebellar foliation defects than that in EED^Math1^ mice. We speculate that it might depend on the Cre mouse lines, where specifically leads to EED deletion starting at different stages and different cell types. It has shown that cerebellar patterning is a complex process that relies on neurogenesis, including the proliferation, cell cycle exit, migration, differentiation of NSCs and progenitor cells, as well as synapse formation and functional maturation. The hGFAP promoter is activated and expressed in radial glial cells (RGCs) from E13.5 onward.^[^ ^21^ ^]^ The *hGFAP‐cre* transgene induces recombination early in CNS embryogenesis and results in the ablation of EED in the precursor cells of cerebellar progenitors, Bergmann glia (BG), associated astrocytes, and cortical barrel‐forming neurons. This broad and early deletion of EED led to a severely malformed cerebellum, likely due to disrupted functions in each of the affected cell types, all of which exhibited morphological defects.

During embryonic neurogenesis, the cerebellar neuroepithelium (*hGFAP^+^*) is primarily divided into two compartments: the upper rhombic lip (*Atoh1^+^*) and the cerebellar ventricular zone (*Ptf1a^+^*), which give rise to glutamatergic neurons (e.g., granule cells) and GABAergic neurons (e.g., Purkinje cells), respectively.^[^ ^22^ ^]^ *Math1*(*Atoh1*) is expressed in the precursors of the external granule layer (EGL) in the rhombic lip beginning at E14.5, and later in the EGL of the developing cerebellum. *Math1* expression ceases during the inward migration of the granule cells to the IGL.^[^ ^23^ ^]^ Since *Math1‐Cre* expression is restricted to GCPs and CGNs among cerebellar cell types, we assume that the persistent EED signal in the cerebellum of the conditional knockouts arises from other cell types, such as Purkinje cells, astrocytes, and microglia.^[^ ^24^ ^]^ Consequently, cerebellar foliation defects are more pronounced in EED^hGFAP^ mice than in EED^Math1^ mice, with motor defects also being more severe in EED^hGFAP^ mice. We hope future studies requiring CreERT2 systems will facilitate controlled activation or deactivation of CRE expression at specific times, enabling precise genetic manipulation at targeted developmental stages.

Dysregulation of PRC2 components has been implicated in multiple human developmental diseases, neurological disorders, and cancers. Loss of function variants in individual PRC2 subunits, EED, SUZ12, or EZH2, causes human Cohen‐ Gibson or Weaver‐like syndrome, a rare congenital disorder characterized by increased growth, macrocephaly, and variable intellectual disability.^[^ ^25^ ^]^ Intriguingly, studies indicated that a cohort of patients with Weaver‐like syndrome exhibits structural defects and neuronal migration anomalies in the brain.^[^ ^26^ ^]^ This is in agreement with our present finding that EED ablation led to disruption of cerebellar organization, including disrupted proliferation and migration of GCPs, and cell apoptosis, which further confirmed the key role of EED in cerebellar development.

### 3.2 EED‐Mediated H3K27me3 Plays an Essential Role in the Repression of a Subset of Genes in Mouse Cerebellum Development

Genes and pathways, including Wnt and TGF‐β/BMP pathways, play crucial roles in cerebellar development.^[^ ^27^ ^]^ Overactivation of Wnt/β‐catenin signaling resulted in premature differentiation of GCPs and mice presented with significantly impaired motor coordination and ataxia.^[^ ^28^ ^]^ The proliferative aspects of Wnt‐induced self‐renewal may be driven by such genes as *Ccnd1*, *Ccnd2*. In our study, EED deletion led to increased expression of *Ccnd2*, a key player in the Wnt signaling pathway,^[^ ^29^ ^]^ and decreased expression of *Frzb*, a Wnt antagonist. This aligns with previous findings that EED/PRC2‐mediated histone methylation activates Wnt signaling during CNS myelination.^[^ ^20a^ ^]^ Our data imply that EED is a pivotal epigenetic modifier that requires appropriate levels of Wnt signaling to balance GCPs proliferation and differentiation in cerebellar development (Figures S10 and S11, Supporting Information). Alder and colleagues showed that BMPs, members of the TGF‐β superfamily, are critical regulators of cerebellar granule neuron specification.^[^ ^30^ ^]^ Here, we report that the TGF‐β/BMP signaling pathway is deregulated in EED‐deleted GCPs (Figure S10, Supporting Information), which is consistent with previous reports that inactivation of PRC2 leads to downregulation of the genes associated with loss of H3K27me3.^[^ ^31^ ^]^ These data suggest that EED governs cerebellum development in part through an H3K27me3‐dependent mechanism and precise regulation of TGF‐β/BMP signaling is required for the proper formation of the cerebellum.

Our results indicate that the knockdown of H19 partially rescues the proliferation, migration, and motor deficit in *Eed* cKO mice. Combined RNA‐seq and ChIP assays clearly demonstrated that, in addition to H19, EED may also directly regulate dozens of other genes and pathways that merit further investigation regarding their regulatory mechanisms in GCPs. Taken together, we conclude that EED is an essential regulator of cerebellar neurogenesis and development, and the transcriptome‐wide gene expression dysregulation induced by EED depletion ultimately results in reduced GCPs proliferation, migration, increased apoptosis, and cerebellum hypoplasia. Furthermore, our behavioral tests indicate that cerebellar hypoplasia in *Eed* cKO mice is associated with developmental delay and impaired motor coordination.

H3K27me3, stimulated by PRC2 or removed by histone lysine demethylase enzymes *Kdm6a/b*, at the promoters and distal regulatory sites of genes to permanently silence gene expression in postmitotic neurons, including in the cerebellum.^[^ ^6^ ^]^ Deletion of EZH2 in cerebellar precursors results in reduced precursor proliferation and Purkinje cell number, and EZH2‐deficient mice exhibit severe cerebellar hypoplasia.^[^ ^32^ ^]^ Ablation of both EZH1 and EZH2 specifically derepresses gene expression and impairs the survival of adult Purkinje cells, resulting in cerebellar degeneration.^[^ ^7^ ^]^ *Kdm6b* activates gene expression by inducing H3K27me3 demethylation and recruiting histone H3K4 methyltransferases to gene promoters.^[^ ^33^ ^]^ *Kdm6b* expression is significantly upregulated in differentiating granule neurons, and knockout of *Kdm6b* impairs cerebellar growth.^[^ ^33^, ^34^ ^]^ Here in our study knockout of EED in NSCs or GCPs leads to reduced H3K27me3 and depression of genes which are associated with cerebellum development or hypoplasia.

H3K27me3 homeostasis is important for the central nervous system. Abnormal H3K27me3 causes many neurological diseases.^[^ ^35^ ^]^ Recent studies have reported that elevated H3K27me3 has been involved in the pathogenesis of ataxia‐telangiectasia (A‐T) caused by ATM protein deficiency. A‐T postmitotic neurons have increased PRC2 and undergo cell death, indicating that controlling the expression of H3K27me3 is crucial for neuronal function and survival.^[^ ^36^ ^]^ Thus, in addition to its role in orchestrating topographic migration and connectivity of hippocampus neurons^[^ ^5^ ^]^ and in neuron survival,^[^ ^36^ ^]^ our study indicates that EED regulates a subset of genes underlying the appropriate proliferation and differentiation of neural precursor cells dependent on H3K27me3. What's more, loss of EED caused mice a growth defect and cerebellar hypoplasia.

### 3.3 EED/PRC2 and Non‐Coding RNA H19 Form a Loop to Promote GCPs Differentiation

Our data identified EED/PRC2 complex contains methylating histone H3 and it has also been shown to interact with histone deacetylases. These results provide biochemical mechanisms by which the EED/PRC2 complex may establish heritable epigenetic states leading to long‐term gene repression in vivo. In heart maturation, gene upregulation in *Eed* cKO was not coupled with loss of H3K27me3, and rather the activating histone mark H3K27ac increased.^[^ ^9^ ^]^ In addition to as a repressor dependent on H3K27me3, we also found that EED/PRC2 is required to suppress H3K27ac, and that loss of this function leads to abnormal H3K27ac accumulation. Our data showed H3K27ac is higher at the promoter of *Ctcf* sites in *Eed* cKO mice cerebellum to promote H19 transcription.

In the past decade, several studies have demonstrated that noncoding RNAs have crucial roles in both normal cerebellar development and pathogenesis.^[^ ^37^ ^]^ H19 and *Igf2* are imprinted reciprocally by methylation of specific alleles within the *Igf2*/H19 ICR.^[^ ^38^ ^]^ In both humans and mice, DNA methylation of *Igf2*/H19 ICR correlates with the weight of the cerebellum.^[^ ^39^ ^]^ Utilizing gene expression and chromatin accessibility assays in mouse zygotes and morula embryos, Inoue and colleagues revealed that H3K27me3 is responsible for maternal allele inaccessibility at DNA hypomethylated regions.^[^ ^40^ ^]^ Ectopic removal of H3K27me3 caused the expression of the maternal allele.^[^ ^41^ ^]^ Furthermore, recent studies demonstrated that depletion of EED, a core subunit of PRC2, in oocytes results in loss of maternal H3K27me3, ectopic maternal Xist expression, and aberrant maternal XCI in embryos of both sexes.^[^ ^41^, ^42^, ^43^ ^]^ Here, we provide novel evidence showing that in *Eed* cKO cerebellum the level of H3K27me3 is decreased in the ICR of H19 and the expressions of H19 are upregulated. EED can directly bind to the ICR of H19 and knockdown of H19 can partially rescue the proliferation, migration, and motor deficit in *Eed* cKO mice. Therefore, we boldly speculate that the enrichment of maternal H3K27me3 reduces in the ICR after depletion of EED, then resulting in increasing the expression of maternal H19. To differentiate paternal and maternal information using SNPs, heterozygous F1 generation mice from different strains are required. As described by Guo F et al. ^[^ ^44^ ^]^ they used paternal strain 129sv mice and maternal strain C57BL/6J mice for crossbreeding, then downloaded SNP information from the Sanger Institute to analyze the differences in DNA methylation and chromatin accessibility between paternal and maternal alleles. Unfortunately, our data cannot distinguish between paternal and maternal allele reads, which is one of limitations in this study. Future studies are needed to reveal this mechanism.

The control of transcription for the H19 gene appears to be complicated. H19 was demonstrated to regulate EZH2‐mediated H3K27me3 in the setting of cancer cells.^[^ ^45^ ^]^ We proposed PRC2 subunit EED may be also controlled by H19 in the cerebellum. Our data showed a positive correlation between H19 expression and EED activation. Interestingly, EED deletion reversely increases the expression of H19, which pointed out that EED regulates the expression of H19 and functions as a negative feedback mechanism in cerebellum development.

Previous studies have identified higher levels of H19 mRNA observed in the cerebellum compared with the frontal cortex.^[^ ^46^ ^]^ It will be of great interest to explore the potential functional H19 in the development of the cerebellum. We first demonstrated that H19 inhibited proliferation and enhanced apoptosis of GCPs in vivo. H19 upregulation was responsible for the *Eed* cKO cerebellar hypoplasia on motor development and function. Cerebellar DNA methylation at CTCF‐binding sites, upstream of H19, shows a negative relationship with cerebellum mass, it could be interesting to investigate the cooperation action among EED, H19, and DNA methylation.

In summary, our results not only highlight the EED is required for normal cerebellar development but also point to the fact that lack of EED in GCPs could result in cerebellar morphological deficits through upregulation of non‐coding RNA H19. Our study indicates that EED functions as a mediator of mice brain development by repressing multiple downstream pathways, including non‐coding RNA H19, a finding that adds to the complexity of EED biology. Moreover, we demonstrate that H19 regulates EED‐mediated H2K27me3 expression in the cerebellum. Our studies reveal that EED and H19 form a negative feedback loop to regulate cerebellum development. The genetic cerebellum hypoplasia and motor defects model presented here may open a new avenue through which to model and study the pathogenesis of human neurodevelopmental brain disorders.

## 4 Experimental Section

### Animals

EED^f/f^ mice were described previously.^[^ ^5^ ^]^ The hGFAP‐Cre (Strain #:02 4098, C57BL6 background) was bought from the Jackson lab. The Math1‐Cre (Strain#:01 1104, C57BL6 background) was bought from the Jackson lab. Conditional knockout mice were generated by breeding EED^f/f^ mice with hGFAP‐Cre, or Math1‐Cre transgenic mice. H19 KO mice were provided by the lab of Rong‐Feng Li, Nanjing Medical University. The detailed mouse breeding schematic was as follows. Female H19^+/−^ (H19 Het) mice and male H19^+/−^ (H19 Het) mice to generate male and female H19 ^−/−^ homozygous offspring were used. The wild‐type mice generated during the breeding process were used as controls. Developmental stage, brain region, strain consistency, and housing conditions were carefully controlled. The function of H19 ^−/−^ (H19 KO) in male and female mice was focused on.

Mice were housed in a room at a constant temperature (23 °C) with a regular 12 h light/dark cycle. Mice genotypes were performed by PCR assay using tail genomic DNA. Genotyping primers are listed in Table S1 (Supporting Information). All procedures were approved by the Animal Committee of the Institute of Zoology, Chinese Academy of Sciences, and were conducted according to the guidelines of national ethical regulations for animal care and use in research (IOZ20180044).

### GCPs Cultures

GCPs were purified from the cerebella of P6 mice by using Percoll gradient sedimentation.^[^ ^34^ ^]^ Briefly, cerebella from P6 mice were mechanically triturated into small pieces and then digested at 37 °C for 10 min in trypsin (Invitrogen). The cell suspension was passed through a cell strainer, layered on a step gradient of 35% and 65% Percoll (Sigma), and centrifuged at 2500 rpm for 12 min at room temperature. Purified GCPs were harvested from the 35%/65% interface, washed in PBS, and resuspended in a neurobasal medium with B27 supplements and 2% FBS and then plated in poly‐D‐lysine/laminin–coated plates. For proliferation assay, GCPs were cultured with Human recombinant SHH (75 ng mL^−1^; Applied Stem Cell). For cell survival assay, GCPs were differentiated without Human recombinant SHH into postmitotic CGNs.

### LncRNA Smart Silencer (siRNA) Transfection

LncRNA Smart Silencer transfection in GCP cells was performed by Lipofectamine RNAiMAX(Invitrogen), lncRNA H19 Smart Silencer was purchased from RiboBio(lnc3170930111639). The sequence of siH19 was used as follows: 5′‐AAGGTATAGCTGGCAGCAGT‐3′, 5′‐GTTAGCAAAGGAGACATCGT‐3′, 5′‐TCCGGTGTGATGGAGAGGAC‐3′, 5′‐GAACCCTCAAGATGAAAGA‐3′, 5′‐AGGGATTTTACAGCAAGGA‐3′, 5′‐GGATCCAGCAAGAACAGAA‐3′. lncRNA Smart Silencer NC was purchased from RiboBio (lnc3N0000001‐1‐5).

### BrdU Labeling and Cell Migration

BrdU (Sigma–Aldrich) was intraperitoneally administered to P7 mice with a dosage of 100 mg per gram of body weight. For the analysis of cell proliferation experiments, 2 h after injection, mice were sacrificed, perfused in 4% paraformaldehyde (PFA) and cerebella were taken out for fixation. For the analysis of newborn cerebellar granule cells (CGCs), mice (P7) were injected with BrdU and were kept for another 2 days until they were killed at P10 for staining. For the analysis of BrdU incorporation cell migration experiments,^[^ ^47^ ^]^ mice (P7) received one 50 ug g^−1^ dose of BrdU via intraperitoneal injection and were sacrificed after 7 days.^[^ ^20a^ ^]^ BrdU incorporation was measured by immunofluorescence staining as described below.

### Histology and Immunostaining

Cerebella from P0‐ P21 mice were collected and tissues were fixed with 4% PFA in PBS overnight at 4 °C and then dehydrated in 30% sucrose in PBS at 4 °C. Following dehydration, cerebella were cut into 40 µm thick floating sagittal sections using a freezing microtome (Leica CM 1950). The neonatal cerebella were cut into 15–20 µm sections mounting on Superfrost Plus microscope slides (Thermo Fisher Scientific). Sections with cresyl violet and imaged them for morphology were stained. Size measurements were performed using ImageJ to quantify the area from midline sections (for cerebellar vermis size) of each of the three mice.

For immunostaining of sections or isolated cells, sections or coverslips were washed in PBS for 10 min three times, fixed with 4% PFA/PBS for 15 min, permeabilized with 0.5% Triton X‐100 for 10 min, and blocked in a solution of 0.5% Triton X‐100, 3% Bovine Serum Albumin (BSA) at room temperature for 1 h. For BrdU staining, sections or coverslips were treated with 1n HCl for 30 min at 37 °C. Primary antibodies were incubated in the 3% BSA and 0.5% Triton X‐100 at 4 °C overnight. The primary antibodies used in the study as follows: rabbit anti‐Calbindin (1:5000, D28K‐300, Swant), rabbit anti‐Pax6(1:1000, 901031, Biolegend), rabbit anti‐Ki67 (1:1000, RM‐9106‐S, Thermo), rat anti‐BrdU (1:1000, ab6326, Abcam), mouse anti‐NeuN (1:500, MAB377, Millipore), rabbit anti‐EED (1:200, ab126542, Abcam). Sections or coverslips were washed in PBS and incubated with Alexa Fluor‐conjugated secondary antibodies (Invitrogen) for 1 h with a concentration of 1: 500 at room temperature. The sections or coverslips were washed three times and mounted on slices using an adhesion antifade medium. Confocal images were acquired with a confocal laser‐scanning microscope and were analyzed with ImageJ.

### RNA Extraction and qRT‐PCR

Cerebella tissues were dissected rapidly from mice, frozen in liquid nitrogen, and stored at −80 °C for further processing. Total RNA was lysated from frozen tissues and cultured cells using 1 mL Trizol reagent according to the manufacturer's manual (Invitrogen). After extraction, RNA was reverse transcribed into cDNA with TransScript One‐Step gDNA Removal and cDNA Synthesis Kit (TRANS). The cDNA was analyzed by quantitative real‐time PCR (qPCR) using SYBR *Premix Ex Taq* (Tli RNaseH Plus) (Takara). Each sample was run in triplicate. mRNA levels were normalized to values for GAPDH. The analysis of qPCR used the 2^−ΔΔCT^ method. The following primer sequences are listed in Table S1 (Supporting Information).

### Western Blotting

Western blotting was performed as published.^[^ ^48^ ^]^ The cerebella were rapidly removed from mice under ice‐cold saline and lysed in ice‐cold RIPA buffer (Beyotime, P0013B) with a protease inhibitor cocktail (Roche). The protein concentration was measured by using a BCA protein assay kit (Biomed, PP0102). Samples were boiled, chilled on ice, and then centrifuged at 13000 rpm/3 min at 4 °C. Proteins were run in 8–15% SDS‐PAGE gels (Bio‐Rad) and transferred to PVDF membranes (Millipore). The membrane was blocked in 5% milk in TBST (TBS+0.05% Tween‐20) for 1 h at room temperature and incubated with primary antibodies at 4 °C overnight. On the second day, membranes were then washed and incubated with HRP‐coupled goat anti‐rabbit or HRP‐coupled goat anti‐mouse secondary antibody for 1 h at room temperature. The following dilutions of the primary antibodies were used: rabbit anti‐H3(1:1000, 4499, Cell Signaling), mouse goat anti‐EED (1:2000, ab240650, Abcam), mouse anti‐β‐Actin (1:10 000, A5441, Sigma), rabbit anti‐H3K27ac (1:5000, ab4729, Abcam), rabbit anti‐H3K27me3 (1:5000, 07–449, Millipore), anti‐GAPDH(1:5000, BE0034‐100, EASYBIO), anti‐β‐Actin(1:5000, BE0033‐100, EASYBIO). Protein bands were visualized by enhanced chemiluminescence reagent (ECL, Pierce) and quantified using ImageJ software (NIH, USA). Quantification of protein was normalized to GAPDH, β‐Actin or histone 3 in the same lane.

### RNA‐Seq and Bioinformatics Analyses

RNA‐seq libraries from WT and *Eed* cKO cerebellar at P14 were prepared using Illumina RNA‐Seq Preparation Kit and sequenced by HiSeq 2500 sequencer. High‐quality reads of RNA‐seq were quantified using Salmon (v.1.1.0) with the parameter –validate Mappings –gcBias″ and gene expression matrix was generated by tximport (v1.14.2).^[^ ^49^ ^]^ Differential gene expression analysis was conducted using DESeq2 (v1.26.0).^[^ ^50^ ^]^ Only transcripts that showed more than 1.5‐fold differential expression and *p*‐value < 0.05 compared to control were subjected to relevance network analysis. Gene ontology analysis was performed by dropping the up and down‐regulated gene lists into the database for annotation, visualization, and integrated discovery programs.^[^ ^51^ ^]^ For custom gene sets, GSEA was performed for the analysis of gene expression changes. Normalized enrichment score reflects the degree to which the gene set is overrepresented at the top or bottom of a ranked list of genes. Gene ontology biological processes are listed in Tables S2 and S3 (Supporting Information).

### Chromatin Immunoprecipitation (ChIP)

ChIP was performed as described earlier. In brief, cerebellar tissues isolated from EED WT and cKO were fixed with 1% formaldehyde (Sigma–Aldrich) to the culture medium for 10 min at room temperature. After washing with cold PBS, tissues were suspended in 1 mL of cold cell lysis buffer (5 mm PIPES, pH 8.0, 85 mm KCl, 0.5% NP‐40, 1×proteinase inhibitor cocktail). Lysates were centrifuged, resuspended again in cell lysis buffer, and then collected nuclei. Nuclei were lysed at room temperature with 500 µL of nuclei lysis buffer (50 mm Tris, pH 8.1, 10 mm EDTA, 1% SDS, 1×protease inhibitor cocktail). DNA was sonicated for 30 min at 4 °C using a sonicator to achieve fragments of sizes ranging from 200 to 500 bp and then protein–DNA complexes were incubated with protein bound antibodies (10 µg) at 4 °C overnight. Antibodies used were: normal rabbit IgG (ChIP grade, 2729, Cell Signaling), mouse to EED (ChIP grade, 61203, Active Motif), rabbit polyclonal to H3K27me3 (ChIP grade, 07–449, Millipore), rabbit polyclonal to H3K27ac (ChIP grade, ab4729, Abcam). After incubation, chromatin pulled down by the Protein A–bound antibodies were washed sequentially two times each in IP dilution buffer, TSE‐500 solution (0.1% SDS, 1% Triton X‐100, 2 mm EDTA, 20 mm Tris, pH 8.1, 500 mm NaCl), Li/Cl wash solution (100 mm Tris, pH 8.1, 300 mm LiCl, 1% NP‐40, 1% deoxycholic acid), and 1× Tris‐EDTA buffer (TE) for 10 min at 4 °C. Protein–DNA complexes were eluted from beads twice with IP elution buffer (50 mm NaHCO3 and 1% SDS) for 15 min at room temperature with rotation. Formaldehyde‐induced protein–DNA cross‐linking was heat reversed by incubation at 65 °C overnight. DNA was extracted with phenol/chloroform/isoamyl alcohol (25:24:1) isolations and precipitated with two volumes of 100% ethanol and 10 µg linear acrylamide at −20 °C overnight. Purified DNA fragments were resuspended in nuclease‐free water. The following primer sequences are listed in Table S1(Supporting Information).

### Analysis of ChIP‐Seq

DNA libraries generated from ChIP‐DNA and input‐DNA were deep‐sequenced using HiSeq single‐end 50 bp. After trimming with trimmomatic (v.0.36), high‐quality reads were mapped to the mm10 genome with bowtie 2(v2.4.1) using default parameters.^[^ ^52^ ^]^ Peaks were called with macs2 with default parameters relative to the input sample.^[^ ^53^ ^]^ Manorm (v.1.2.0) was then used for quantitative comparison of ChIP‐Seq data.^[^ ^54^ ^]^ Increased and decreased H3K27ac or H3K27me3 enrichment were defined by M value (log2(fold‐change)) > 0.585 and M value < −0.585 with and *p*‐value < 0.05 (1.5‐fold change), respectively. Peak annotation was performed using ChIPseeker (v.1.22.1) at the gene level and promoter regions were defined as ± 1000 bp of TSS.^[^ ^55^ ^]^ Coverage density plots were done with deeptools (v. 3.4.0).^[^ ^56^ ^]^ Peak annotation genes are listed in Table S4 (Supporting Information) (H3K27me3 peaks) and Table S5 (H3K27ac peaks) (Supporting Information).

### Behavioral Tests

For the open field test, a 30 × 30 open box made from clear plexiglass was used. Each subject mouse will be given 5 min (5 min in optogenetic experiments) to freely explore the box. The total travel distance and mean speed were recorded.

Motor coordination and learning were assessed on a rotating rod as described previously,^[^ ^57^ ^]^ when the mice reached 14–21 days of age. The latency to fall for any particular day was calculated as the mean of 2 trials.

### Statistical Analysis

The cerebellar immunofluorescence images were imaged with confocal scanning. The thickness of cell layers, the cerebellum area, the number of BrdU^+^ and Ki67^+^ cells were statistically analyzed using Image J per unit area. Quantification of the number of TUNEL‐positive cells per square unit (mm^2^) was used.^[^ ^58^ ^]^ All of the experiments were performed in triplicates. Statistics were used GraphPad prism software and data was analyzed using two‐tailed unpaired Student's *t*‐test and ANOVA to compare the differences. Data were shown as mean ± SEM. Differences were considered significant if *p *< 0.05. *p*‐values are denoted by asterisks: ^*^ *p *< 0.05; ^**^ *p *< 0.01; ^***^ *p *< 0.001.

## Conflict of Interest

The authors declare no conflict of interest.

## Author Contributions

P.‐P. L., X.H., and X.L. contributed equally to this paper. C.M.L., Y.G.Y., and P.P.L., conceptualized and design the project, did the collection, interpretation, assembly of data, analyzed the data, and wrote the final manuscript, gave final approval of manuscript; X.H., X.L, S.K.D, Y.J.X., H.Z.D., and Z.Q.T., did collection and assembly of data.

## Supporting information



Supporting Information

Supporting Information

Supporting Information

Supporting Information

Supporting Information

Supporting Information

## Data Availability

The data that support the findings of this study are available from the corresponding author upon reasonable request.

## References

[1] P. L. Strick , R. P. Dum , J. A. Fiez , Annu. Rev. Neurosci. 2009, 32, 413. 19555291 10.1146/annurev.neuro.31.060407.125606

[2] Y. Yang , T. Yamada , A. Bonni , Handbook of the Cerebellum and Cerebellar Disorders, Springer, Berlin 2019, 1.

[3] a) C. Yuen , D. Merico , M. Bookman , J. L Howe , B. Thiruvahindrapuram , R. V. Patel , J. Whitney , N. Deflaux , J. Bingham , Z. Wang , G. Pellecchia , J. A. Buchanan , S. Walker , C. R. Marshall , M. Uddin , M. Zarrei , E. Deneault , L. D'Abate , A. J. Chan , S. Koyanagi , T. Paton , S. L. Pereira , N. Hoang , W. Engchuan , E. J. Higginbotham , K. Ho , S. Lamoureux , W. Li , J. R. MacDonald , T. Nalpathamkalam , et al., Nat. Neurosci. 2017, 20, 602; 28263302 10.1038/nn.4524 PMC5501701

[4] Y. Hirabayashi , N. Suzki , M. Tsuboi , T. A. Endo , T. Toyoda , J. Shinga , H. Koseki , M. Vidal , Y. Gotoh , Neuron 2009, 63, 600. 19755104 10.1016/j.neuron.2009.08.021

[5] P. P. Liu , Y. J. Xu , S. K. Dai , H. Z. Du , Y. Y. Wang , X. G. Li , Z. Q. Teng , C. M. Liu , Stem Cell Rep. 2019, 13, 115. 10.1016/j.stemcr.2019.05.010 PMC6627036 31204298

[6] X. Feng , A. H. Juan , H. A. Wang , K. D. Ko , H. Zare , V. Sartorelli , Development 2016, 143, 1971. 27068104 10.1242/dev.132902 PMC4920161

[7] M. von Schimmelmann , P. A. Feinberg , J. M. Sullivan , S. M. Ku , A. Badimon , M. K. Duff , Z. Wang , A. Lachmann , S. Dewell , A. Ma'ayan , M. H. Han , A. Tarakhovsky , A. Schaefer , Nat. Neurosci. 2016, 19, 1321. 27526204 10.1038/nn.4360 PMC5088783

[8] P. P. Liu , Y. J. Xu , Z. Q. Teng , C. M. Liu , Neuroscientist 2018, 24, 208. 29283025 10.1177/1073858417747839

[9] S. Ai , Y. Peng , C. Li , F. Gu , X. Yu , Y. Yue , Q. Ma , J. Chen , Z. Lin , P. Zhou , H. Xie , T. W. Prendiville , W. Zheng , Y. Liu , S. H. Orkin , D. Z. Wang , J. Yu , W. T. Pu , A. He , Elife 2017, 6, e24570. 28394251 10.7554/eLife.24570 PMC5400508

[10] S. F. Zhang , S. K. Dai , H. Z. Du , H. Wang , X. G. Li , Y. Tang , C. M. Liu , Stem Cell Rep. 2022, 17, 2064. 10.1016/j.stemcr.2022.07.004 PMC9481917 35931079

[11] a) A. Gabory , H. Jammes , L. Dandolo , BioEssays 2010, 32, 473,; 20486133 10.1002/bies.200900170

[12] D. Lleres , B. Moindrot , R. Pathak , V. Piras , M. Matelot , B. Pignard , A. Marchand , M. Poncelet , A. Perrin , V. Tellier , R. Feil , D. Noordermeer , Genome Biol. 2019, 20, 272. 31831055 10.1186/s13059-019-1896-8 PMC6909504

[13] S. Ghafouri‐Fard , M. Esmaeili , M. Taheri , Biomed. Pharmacother. 2020, 123, 109774. 31855739 10.1016/j.biopha.2019.109774

[14] E. L. Wakeling , F. Brioude , O. Lokulo‐Sodipe , S. M. O'Connell , J. Salem , J. Bliek , A. P. Canton , K. H. Chrzanowska , J. H. Davies , R. P. Dias , B. Dubern , M. Elbracht , E. Giabicani , A. Grimberg , K. Gronskov , A. C. Hokken‐Koelega , A. A. Jorge , M. Kagami , A. Linglart , M. Maghnie , K. Mohnike , D. Monk , G. E. Moore , P. G. Murray , T. Ogata , I. O. Petit , S. Russo , E. Said , M. Toumba , Z. Tumer , Nat. Rev. Endocrinol. 2017, 13, 105. 27585961 10.1038/nrendo.2016.138

[15] a) Z. Wang , X. J. Zhang , Y. X. Ji , P. Zhang , K. Q. Deng , J. Gong , S. Ren , X. Wang , I. Chen , H. Wang , C. Gao , T. Yokota , Y. S. Ang , S. Li , A. Cass , T. M. Vondriska , G. Li , A. Deb , D. Srivastava , H. T. Yang , X. Xiao , H. Li , Y. Wang , Nat. Med. 2016, 22, 1131; 27618650 10.1038/nm.4179 PMC5053883

[16] O. Deevy , A. P. Bracken , Development 2019, 146, dev181354. 31575610 10.1242/dev.181354 PMC6803372

[17] J. Wang , L. Yang , C. Dong , J. Wang , L. Xu , Y. Qiu , Q. Weng , C. Zhao , M. Xin , Q. R. Lu , Sci. Adv. 2020, 6, eaaz6477,. 32851157 10.1126/sciadv.aaz6477 PMC7423366

[18] B. Sun , E. Chang , A. Gerhartl , F. G. Szele , Cereb Cortex 2018, 28, 1369,. 29415247 10.1093/cercor/bhx289 PMC6093351

[19] M. Zervas , S. Blaess , A. L. Joyner , Curr. Top. Dev. Biol. 2005, 69, 101. 16243598 10.1016/S0070-2153(05)69005-9

[20] a) E. Sanchez‐Ortiz , W. Cho , I. Nazarenko , W. Mo , J. Chen , L. F. Parada , Genes Dev. 2014, 28, 2407; 25367036 10.1101/gad.246603.114 PMC4215185

[21] a) L. Zhuo , M. Theis , I. Alvarez‐Maya , M. Brenner , K. Willecke , A. Messing , Genesis 2001, 31, 85; 11668683 10.1002/gene.10008

[22] a) K. Leto , M. Arancillo , E. B. E. Becker , A. Buffo , C. Chiang , B. J. Ding , W. B. Dobyns , I. Dusart , P. Haldipur , M. E. Hatten , M. Hoshino , A. L. Joyner , M. Kano , D. L. Kilpatrick , N. Koibuchi , S. Marino , S. Martinez , K. J. Millen , T. O. Millner , T. Miyata , E. Parmigiani , K. Schilling , G. Sekerková , R. V. Sillitoe , C. Sotelo , N. Uesaka , A. Wefers , R. J. Wingate , R. Hawkes , Cerebellum 2016, 15, 789; 26439486 10.1007/s12311-015-0724-2 PMC4846577

[23] a) Z. J. Yang , T. Ellis , S. L. Markant , T. A. Read , J. D. Kessler , M. Bourboulas , U. Schüller , R. Machold , G. Fishell , D. H. Rowitch , B. J. Wainwright , R. J. Wechsler‐Reya , Cancer Cell 2008, 14, 135; 18691548 10.1016/j.ccr.2008.07.003 PMC2538687

[24] N. Pan , I. Jahan , J. E. Lee , B. Fritzsch , Cell Tissue Res. 2009, 337, 407. 19609565 10.1007/s00441-009-0826-6 PMC3023111

[25] a) R. Smigiel , A. Biernacka , M. Biela , V. Murcia‐Pienkowski , E. Szmida , P. Gasperowicz , J. Kosinska , G. Kostrzewa , A. A. Koppolu , A. Walczak , D. Wawrzuta , M. Rydzanicz , M. Sasiadek , R. Ploski , J. Hum. Genet. 2018, 63, 517; 29410511 10.1038/s10038-017-0391-x

[26] A. Al‐Salem , M. J. Alshammari , H. Hassan , A. M. Alazami , F. S. Alkuraya , Am J Med Genet A 2013, 161, 225. 10.1002/ajmg.a.35660 23239504

[27] M. F. Roussel , M. E. Hatten , Curr. Top. Dev. Biol. 2011, 94, 235. 21295689 10.1016/B978-0-12-380916-2.00008-5 PMC3213765

[28] A. Lorenz , M. Deutschmann , J. Ahlfeld , C. Prix , A. Koch , R. Smits , R. Fodde , H. A. Kretzschmar , U. Schüller , Mol. Cell. Biol. 2011, 31, 3326. 21690300 10.1128/MCB.05718-11 PMC3147790

[29] Z. Zheng , X. Wang , Y. Zheng , H. Wu , Int. Immunopharmacol. 2024, 126, 111334. 38061119 10.1016/j.intimp.2023.111334

[30] J. Alder , K. J. Lee , T. M. Jessell , M. E. Hatten , Nat. Neurosci. 1999, 2, 535. 10448218 10.1038/9189

[31] R. Margueron , N. Justin , K. Ohno , M. L. Sharpe , J. Son , W. J. Drury , P. Voigt , S. R. Martin , W. R. Taylor , V. De Marco , V. Pirrotta , D. Reinberg , S. J. Gamblin , Nature 2009, 461, 762. 19767730 10.1038/nature08398 PMC3772642

[32] D. Pan , Dev. Cell 2010, 19, 491. 20951342 10.1016/j.devcel.2010.09.011 PMC3124840

[33] X. Shi , Z. Zhang , X. Zhan , M. Cao , T. Satoh , S. Akira , K. Shpargel , T. Magnuson , Q. Li , R. Wang , C. Wang , K. Ge , J. Wu , Nat. Commun. 2014, 5, 5425. 25370275 10.1038/ncomms6425 PMC5232137

[34] R. Wijayatunge , F. Liu , K. B. Shpargel , N. J. Wayne , U. Chan , J. V. Boua , T. Magnuson , A. E. West , Mol. Cell. Neurosci. 2018, 87, 4. 29254825 10.1016/j.mcn.2017.11.005 PMC5828961

[35] M. Jakovcevski , S. Akbarian , Nat. Med. 2012, 18, 1194. 22869198 10.1038/nm.2828 PMC3596876

[36] J. Li , R. P. Hart , E. M. Mallimo , M. R. Swerdel , A. W. Kusnecov , K. Herrup , Nat. Neurosci. 2013, 16, 1745. 24162653 10.1038/nn.3564 PMC3965909

[37] M. E. Hatten , M. F. Roussel , Trends Neurosci. 2011, 34, 134. 21315459 10.1016/j.tins.2011.01.002 PMC3051031

[38] M. Nordin , D. Bergman , M. Halje , W. Engstrom , A. Ward , Cell Prolif 2014, 47, 189. 24738971 10.1111/cpr.12106 PMC6496486

[39] a) R. C. Huang , J. C. Galati , S. Burrows , L. J. Beilin , X. Li , C. E. Pennell , J. van Eekelen , T. A. Mori , L. A. Adams , J. M. Craig , Clin Epigenetics 2012, 4, 21; 23148549 10.1186/1868-7083-4-21 PMC3507742

[40] A. Inoue , L. Jiang , F. L. Lu , T. Suzuki , Y. Zhang , Nature 2017, 547, 419. 28723896 10.1038/nature23262 PMC9674007

[41] a) C. W. Hanna , G. Kelsey , Genome Biol. 2017, 18, 177. 28927436 10.1186/s13059-017-1317-9 PMC5605981

[42] A. Inoue , Z. Y. Chen , Q. Z. Yin , Y. Zhang , Gene Dev 2018, 32, 1525. 30463900 10.1101/gad.318675.118 PMC6295166

[43] C. Harris , M. Cloutier , M. Trotter , M. Hinten , S. Gayen , Z. H. Du , W. Xie , S. Kalantry , Elife 2019, 8, e44258. 30938678 10.7554/eLife.44258 PMC6541438

[44] F. Guo , L. Li , J. Y. Li , X. L. Wu , B. Q. Hu , P. Zhu , L. Wen , F. C. Tang , Cell Res. 2017, 27, 967. 28621329 10.1038/cr.2017.82 PMC5539349

[45] a) X. J. Li , F. Zhou , Y. J. Li , X. Y. Xue , J. R. Qu , G. F. Fan , J. Liu , R. Sun , J. Z. Wu , Q. Zheng , R. P. Liu , Acta Pharmacol Sin 2023, 44, 2479; 37580495 10.1038/s41401-023-01145-z PMC10692088

[46] R. Pidsley , E. Dempster , C. Troakes , S. Al‐Sarraj , J. Mill , Epigenetics 2012, 7, 155. 22395465 10.4161/epi.7.2.18910 PMC3335909

[47] A. Enomoto , N. Asai , T. Namba , Y. Wang , T. Kato , M. Tanaka , H. Tatsumi , S. Taya , D. Tsuboi , K. Kuroda , N. Kaneko , K. Sawamoto , R. Miyamoto , M. Jijiwa , Y. Murakumo , M. Sokabe , T. Seki , K. Kaibuchi , M. Takahashi , Neuron 2009, 63, 774. 19778507 10.1016/j.neuron.2009.08.015

[48] P. P. Liu , G. B. Tang , Y. J. Xu , Y. Q. Zeng , S. F. Zhang , H. Z. Du , Z. Q. Teng , C. M. Liu , Stem Cell Reports 2017, 9, 190. 28602614 10.1016/j.stemcr.2017.05.007 PMC5511050

[49] R. Patro , G. Duggal , M. I. Love , R. A. Irizarry , C. Kingsford , Nat Methods 2017, 14, 417. 28263959 10.1038/nmeth.4197 PMC5600148

[50] C. Soneson , M. I. Love , M. D. Robinson , F1000Res 2015, 4, 1521. 26925227 10.12688/f1000research.7563.1 PMC4712774

[51] W. Huang da , B. T. Sherman , R. A. Lempicki , Nat Protoc 2009, 4, 44. 19131956 10.1038/nprot.2008.211

[52] a) B. Langmead , S. L. Salzberg , Nat Methods 2012, 9, 357; 22388286 10.1038/nmeth.1923 PMC3322381

[53] J. Feng , T. Liu , B. Qin , Y. Zhang , X. S. Liu , Nat Protoc 2012, 7, 1728. 22936215 10.1038/nprot.2012.101 PMC3868217

[54] Z. Shao , Y. Zhang , G. C. Yuan , S. H. Orkin , D. J. Waxman , Genome Biol. 2012, 13, R16. 22424423 10.1186/gb-2012-13-3-r16 PMC3439967

[55] F. Yu , Z. Lu , B. Chen , X. Wu , P. Dong , J. Zheng , J Cell Mol Med 2015, 19, 2617. 26257392 10.1111/jcmm.12655 PMC4627567

[56] F. Ramirez , D. P. Ryan , B. Gruning , V. Bhardwaj , F. Kilpert , A. S. Richter , S. Heyne , F. Dundar , T. Manke , Nucleic Acids Res. 2016, 44, W160. 27079975 10.1093/nar/gkw257 PMC4987876

[57] M. Wohr , J. L. Silverman , M. L. Scattoni , S. M. Turner , M. J. Harris , R. Saxena , J. N. Crawley , Behav Brain Res 2013, 251, 50. 22820233 10.1016/j.bbr.2012.07.024 PMC3979986

[58] F. Mirzamohammadi , G. Papaioannou , J. B. Inloes , E. B. Rankin , H. Xie , E. Schipani , S. H. Orkin , T. Kobayashi , Nat Commun 2016, 7, 12047. 27329220 10.1038/ncomms12047 PMC4917962

