# Supplementary material for: An EED/PRC2‐H19 Loop Regulates Cerebellar Development
Source: Adv Sci (Weinh). 2024 Nov 5;12(1):2403591. doi: 10.1002/advs.202403591 (PMC11714151; doi:10.1002/advs.202403591)
Supplement: Supplementary file 1 — Supporting Information [file ADVS-12-2403591-s002.docx]

Supporting Information

**An EED/PRC2-H19 Loop Regulates Cerebellar Development**

Pei-Pei Liu^1,2,3, *^, Xiao Han^2,4,6*^, Xiao Li^1,2,3, *^, Shang-Kun Dai^1,2,3^, Ya-Jie Xu^1,2,3^, Lin-Fei Jiao^1,2,3^, Hong-Zhen Du^1,2,3^, Lihua Zhao^7^, Rongfeng Li^7,8, #^, Zhao-Qian Teng^1,2,3, #^, Yun-Gui Yang^2,3,4,5,6, #^, Chang-Mei Liu^1,2,3, #^

1.Key Laboratory of Organ Regeneration and Reconstruction, State Key Laboratory of Stem Cell and Reproductive Biology, Institute of Zoology, Chinese Academy of Sciences, Beijing 100101, China

2.University of Chinese Academy of Sciences, Beijing 100049, China.

3.Institute for Stem Cell and Regeneration, Chinese Academy of Sciences, Beijing 100101, China.

4.Key Laboratory of Genomic and Precision Medicine, Collaborative Innovation Center of Genetics and Development, College of Future Technology, Beijing Institute of Genomics, Chinese Academy of Sciences, Beijing 100101, China

5.China National Center for Bioinformation, Beijing 100101, China

6.Sino-Danish College, University of Chinese Academy of Sciences, Beijing 100049, China

7.Jiangsu Key Laboratory of Xenotransplantation, Nanjing Medical University, Nanjing 211166, China

8.Key Laboratory of Targeted Intervention of Cardiovascular Disease, Collaborative Innovation Center for Cardiovascular Disease Translational Medicine, Nanjing Medical University, Nanjing 211166, China

# Correspondence authors: C-M. Liu (liuchm@ioz.ac.cn), Y-G. Yang (ygyang@big.ac.cn), Z-Q. Teng (tengzq@ioz.ac.cn) and R-F Li lirongfeng@njmu.edu.cn

*****These authors contributed equally to this paper.

**Running title:** EED Regulates Cerebellar Development and Motor Behaviors

**Key Words:** EED, PRC2, Cerebellum, Motor movement, H19

**
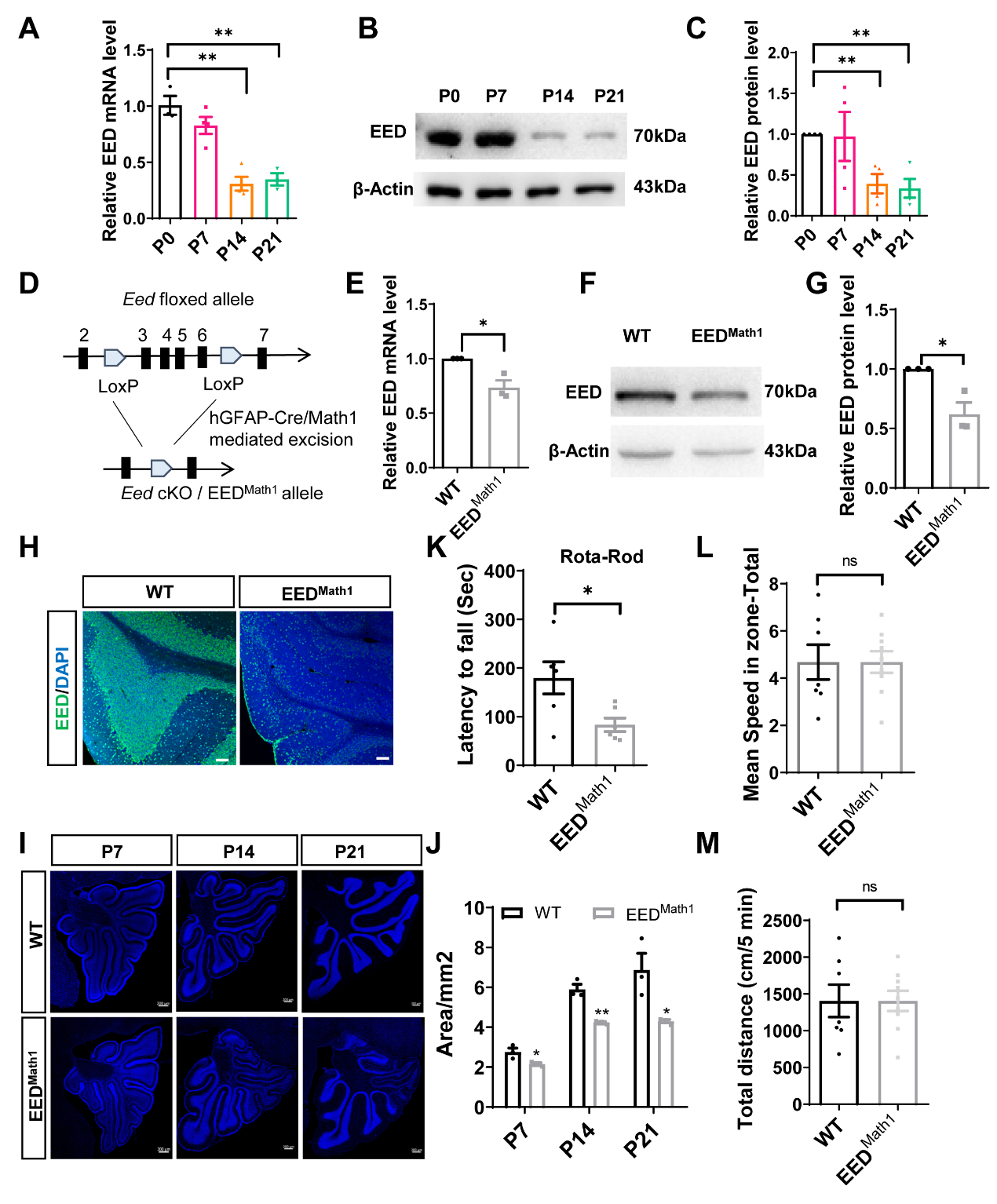
**

**Figure** **S1. EED contributes to cerebellar function**

(A-B) EED expression in cerebellar at different times during postnatal stages was measured by qRT-PCR (A) and western blot (B). ***P*<0.01, n=4, mean ± SEM. (C) Quantification of the density of the EED protein bands by normalization to the intensity of β-actin bands. ***P*<0.01, n=4, mean ± SEM. (D) Model for the generation of *hGFAP-Cre*/*Math1-Cre*-mediated EED knockout mice. (E) qRT-PCR of EED in WT and EED^Math1^ whole cerebellar lysates. **P*<0.05, n=3, mean ± SEM. (F) Western blotting examines the expression of EED in WT and EED^Math1^ whole cerebellar lysates. (G) Quantification of the density of the EED protein bands by normalization to the intensity of β-actin bands. **P*<0.05, n=3, mean ± SEM. (H) Immunostaining of EED(green) in P21 cerebella of WT and EED^Math1^ mice. DNA was stained with DAPI. Scale bars, 50 μm. (I) Representative images of WT and EED^Math1^ cerebellar sagittal sections from P7 through P21. (J) Area of sagittal sections of the cerebellar vermis in WT and EED^Math1^mice from P7 through P21. **P*<0.05, ***P*<0.01, n=3 , mean ± SEM. (K) EED^Math1^ mice showed no change in locomotivity to WT littermate mice in an open field test over a 5-min period. Ns, non-significant. n=7, mean ± SEM. (L) EED^Math1^ mice had similar mean speed to WT littermate mice in open field test over a 5-min period. Ns, non-significant. n=7, mean ± SEM. (M)The mean latency of mice to fall from the rotarod. Note the significant difference between the EED^Math1^ and WT mice. ***P*<0.01, n=7, mean ± SEM.

**
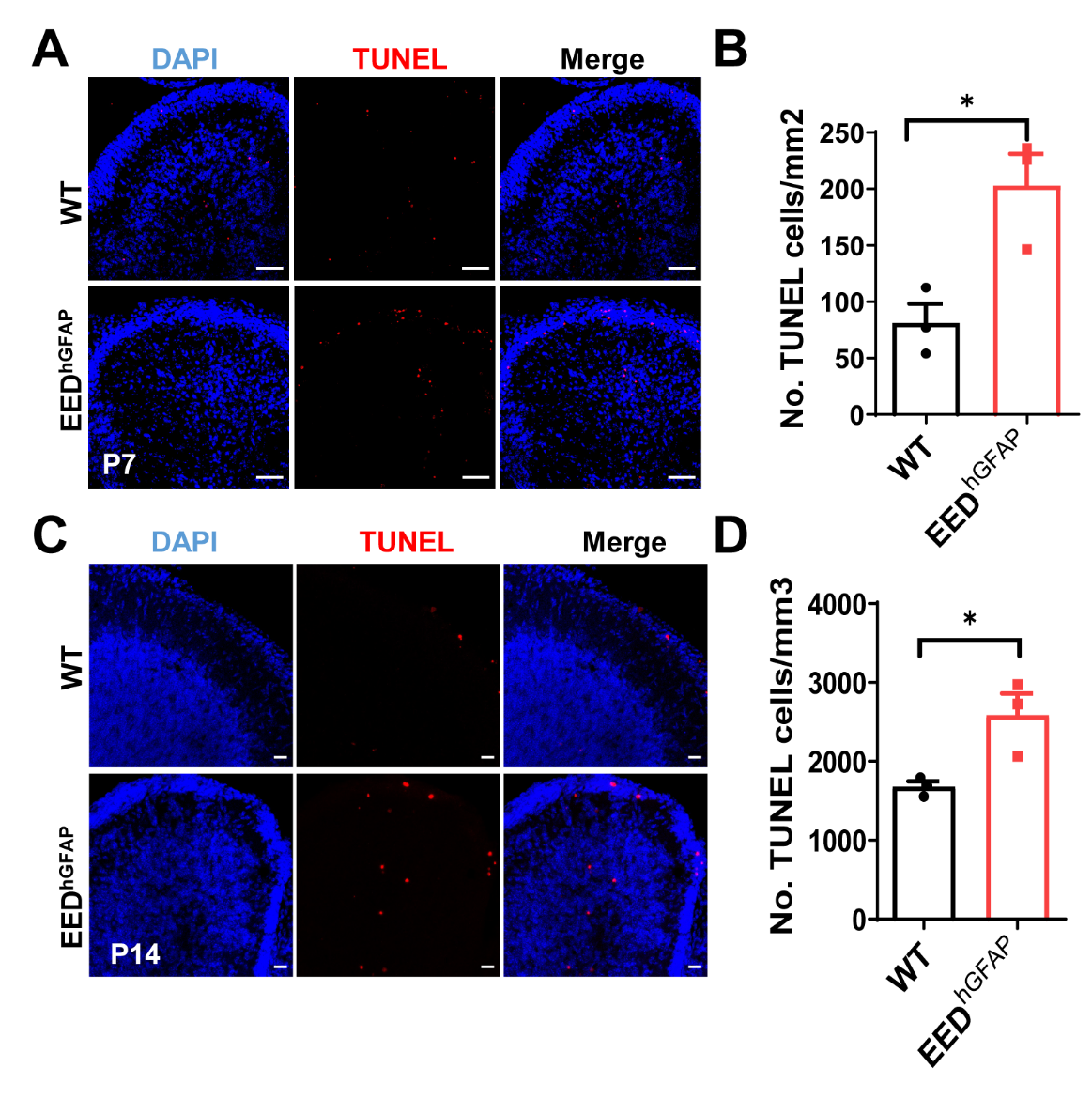
**

**Figure S2. Depletion of EED induces apoptosis of granule cells**

(A) Immunostaining of TUNEL in WT and EED^hGFAP^ mice at P7. Scale bars, 50 μm. (B) The percentage of TUNEL-positive cells (red) per unit area was quantified. **P* < 0.05, n = 3, mean ± SEM. (C) Immunostaining of TUNEL in WT and EED^hGFAP^ mice at P14. Scale bars, 20 μm. (D) The percentage of TUNEL-positive cells (red) per unit area was quantified. **P* < 0.05, n = 3, mean ± SEM.


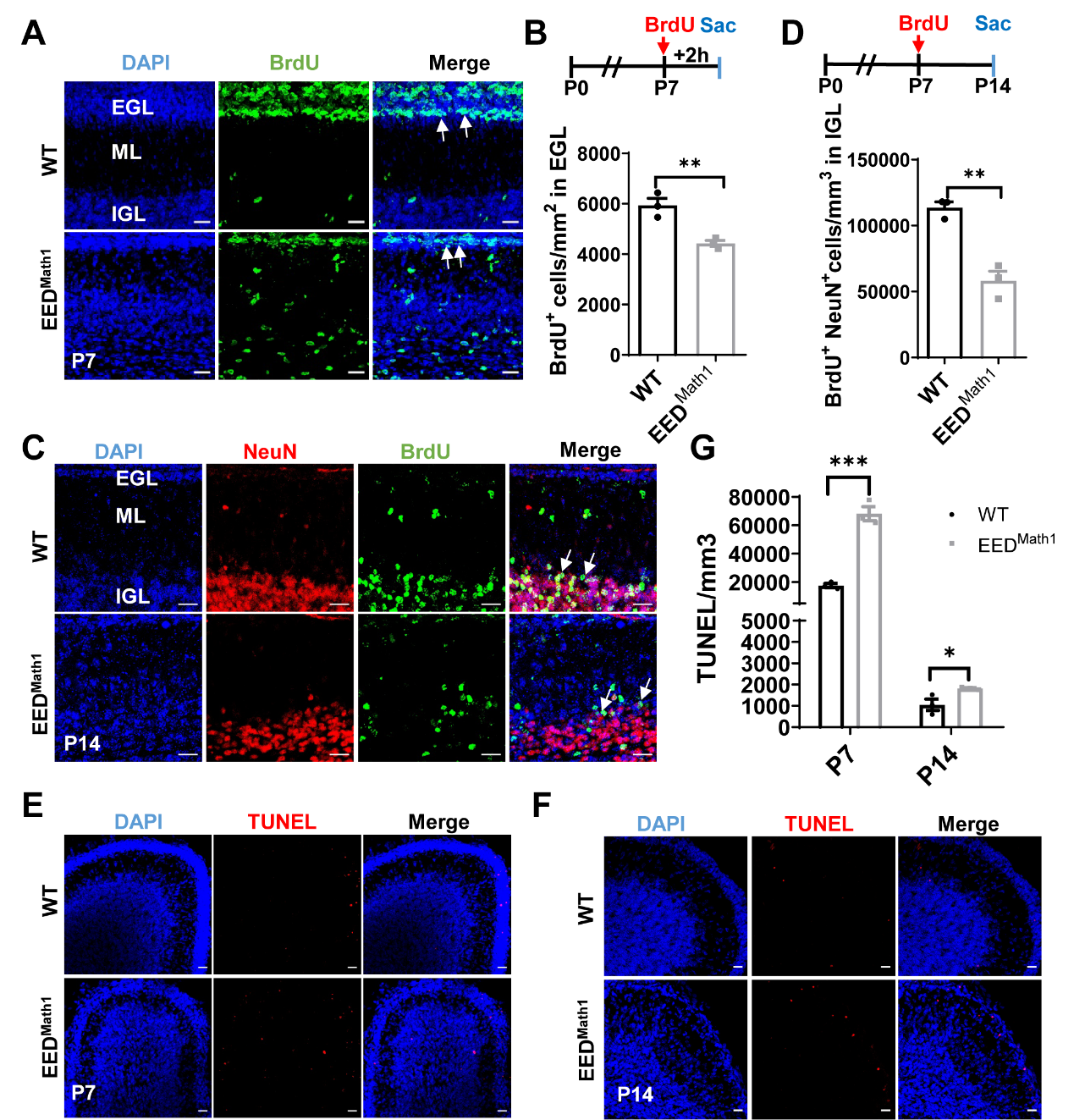


**Figure S3. Defective GCP proliferation and neuronal migration are impaired in EED^Math1^ mice.**

(A) GCPs proliferation in EGL was labeled with anti-BrdU in WT and EED^Math1^ mice at P7. Arrowheads show BrdU-positive cells. Scale bars, 20 μm. (B) GCPs proliferation in EGL was labeled after a 2-h chase following BrdU injection with anti-BrdU in WT and EED^Math1^ mice at P7. Quantification of BrdU-positive cells in the EGL. ***P* < 0.01, n = 3, mean ± SEM. (C) Differentiated granule cells were co-immunolabeled with anti-NeuN antibodies. Arrowheads show decreased BrdU-positive and NeuN^+^ cells in the IGL, indicating impaired migration. Scale bars, 20 μm. (D) Granule neuron migration was tracked in WT and EED^Math1^ mice injected with BrdU at P7 and chased until P14. Quantification of BrdU^+^NeuN^+^cells in the IGL. ***P* < 0.01, n = 3, mean ± SEM. (E) Immunostaining of TUNEL in WT and EED^Math1^ mice at P7. Scale bars, 20 μm. (F) Immunostaining of TUNEL in WT and EED^Math1^ mice at P14. Scale bars, 20 μm. (G) The percentage of TUNEL-positive cells (red) per unit area was quantified at P7 in E and at P14 in F. **P* < 0.05, ****P* < 0.001, n = 3, mean ± SEM.


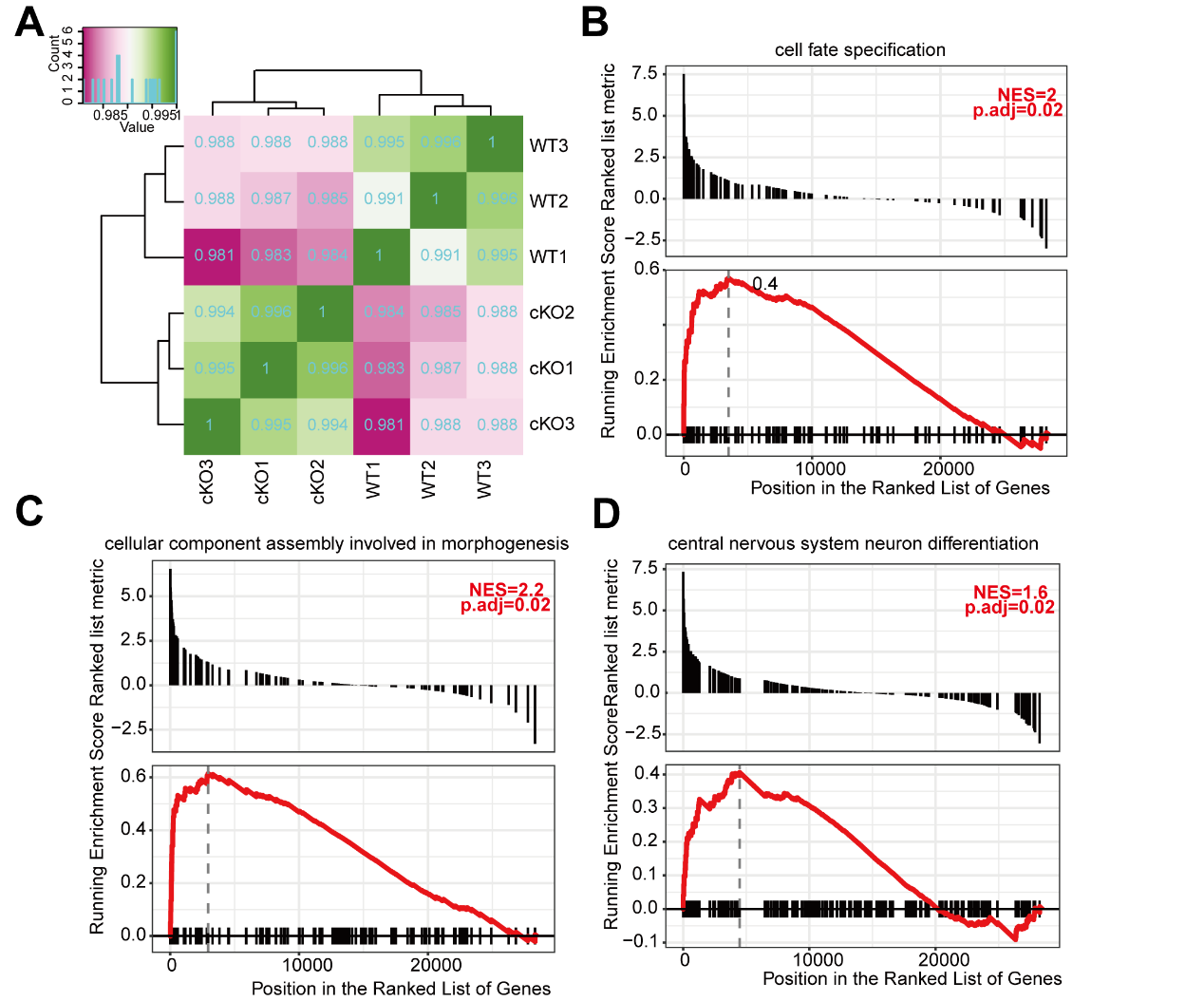


**Figure S4. Loss of EED disturbs gene expression programs that support neural development**

(A) Pearson correlation analysis of WT and *Eed* cKO mice cerebellum RNA-seq samples. (B-D) GSEA of differentially expressed genes enriched in WT or *Eed* cKO cerebellum tissues; NES, net enrichment score.

**
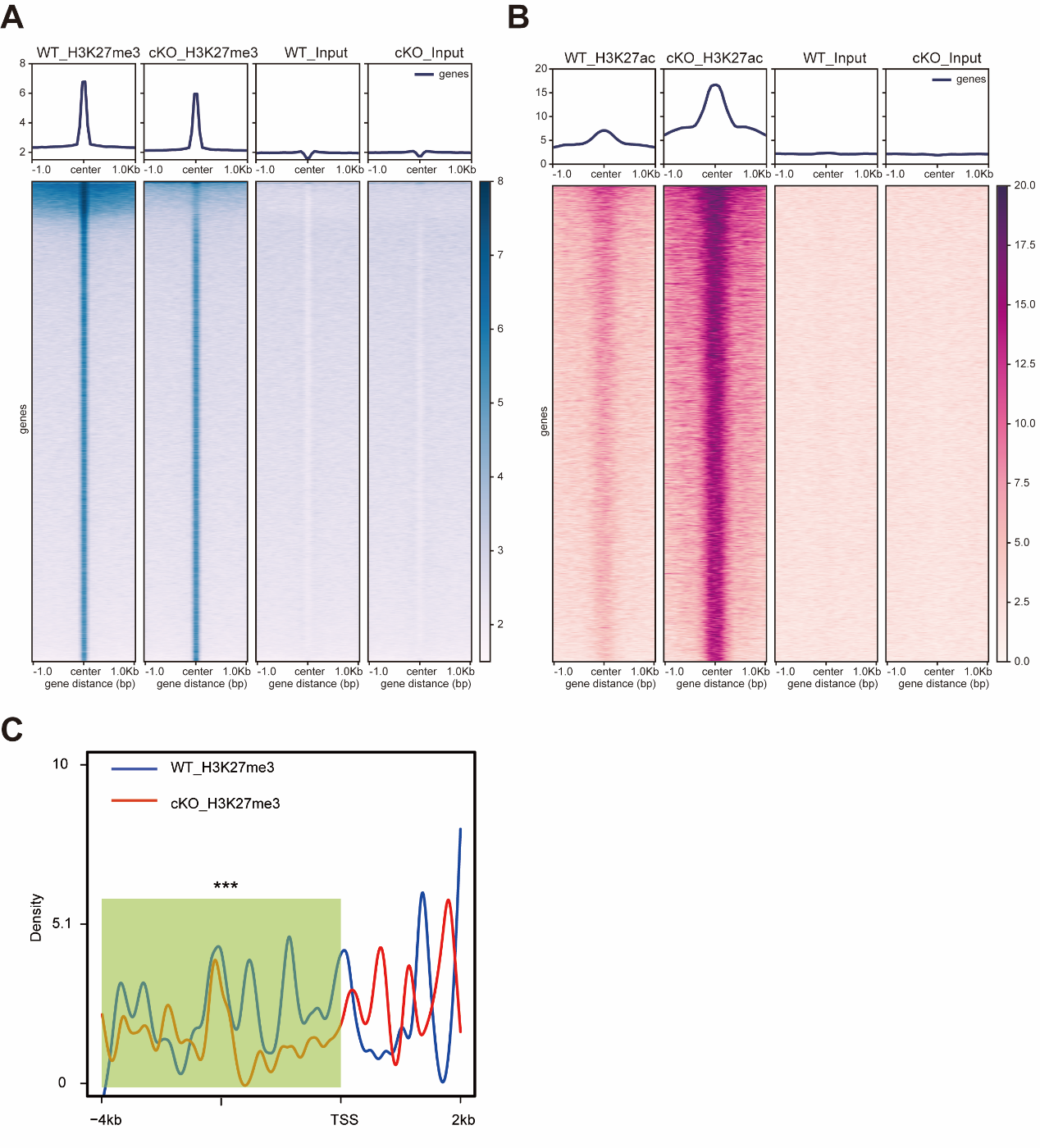
**

**Figure S5. Genome-wide distribution of ChIP-seq signals in WT and *Eed* cKO cerebellum**

(A)Average profiles and heatmaps of H3K27me3 peaks combing all peaks under conditions of WT and *Eed* cKO. (B) Average profiles and heatmaps of H3K27ac peaks combing all peaks under conditions of WT and *Eed* cKO. (C) H3K27me3 density within the 4kb upstream and -2kb downstream of the H19 transcription start site (TSS) in WT and cKO groups. The regions marked in green show the density of H3K27me3 at the 4kb upstream of the H19 gene reduced in *Eed* cKO mice. ****P*<0.001, mean ± SEM.


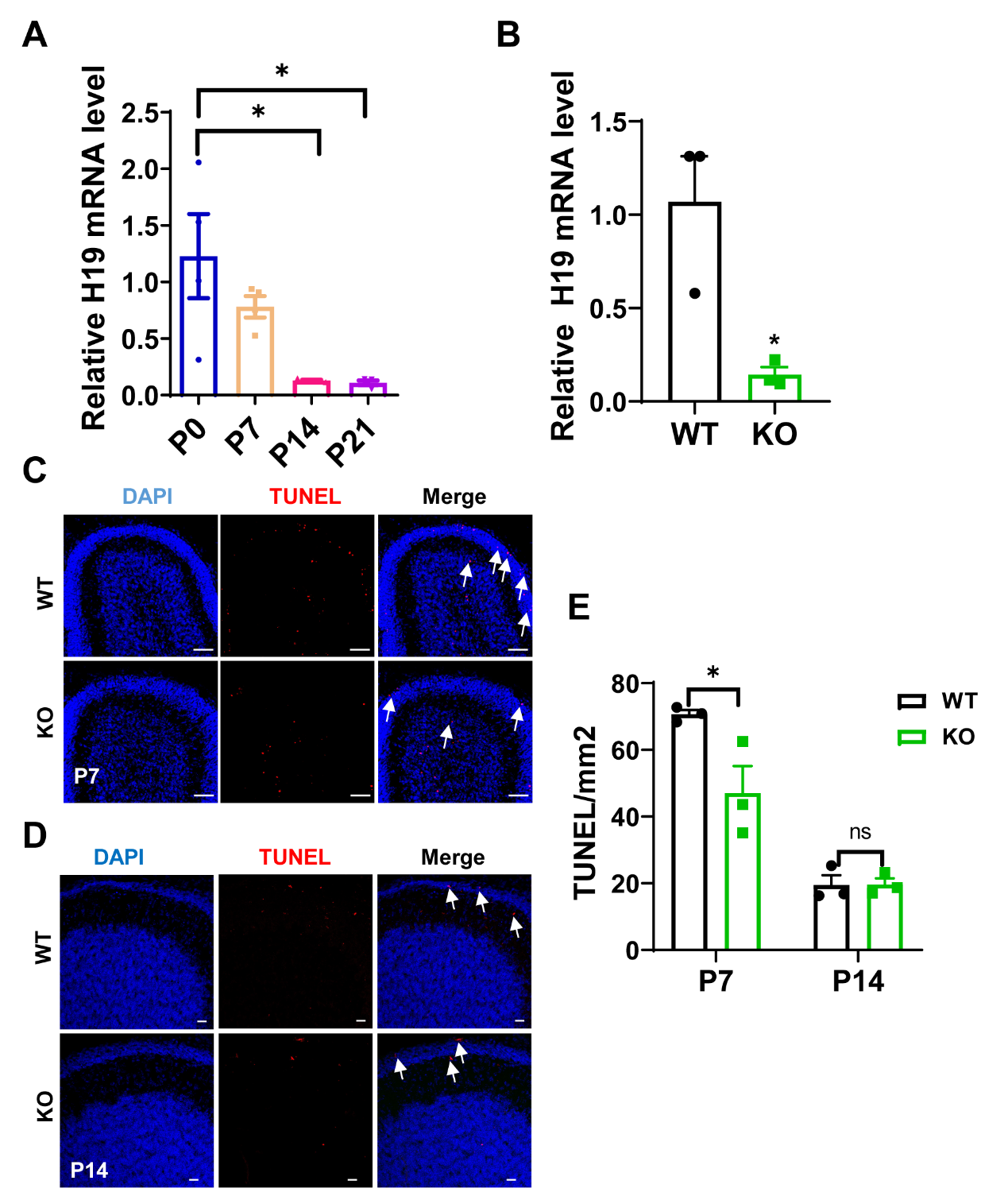


**Figure S6. H19 deletion decreases cell apoptosis**

(A) H19 expression in the cerebellar at different times during postnatal stages from P0 to P21 was measured by qRT-PCR. **P* < 0.05, mean ± SEM. (B) RT-qPCR analysis expression of H19 in WT and H19 KO mice at P14. ***P*<0.01, n=3, mean ± SEM. (C) Immunostaining of TUNEL in WT and H19 KO mice at P7. Scale bars, 50 μm. Arrowheads show TUNEL-positive cells. (D) Immunostaining of TUNEL in WT and H19 KO mice at P14. Scale bars, 20 μm. Arrowheads show TUNEL-positive cells. (E) The percentage of TUNEL-positive cells (red) per unit area was quantified at P7 in E and P14 in F. **P* < 0.05, ns, nonsignificant. n = 3, mean ± SEM.

**
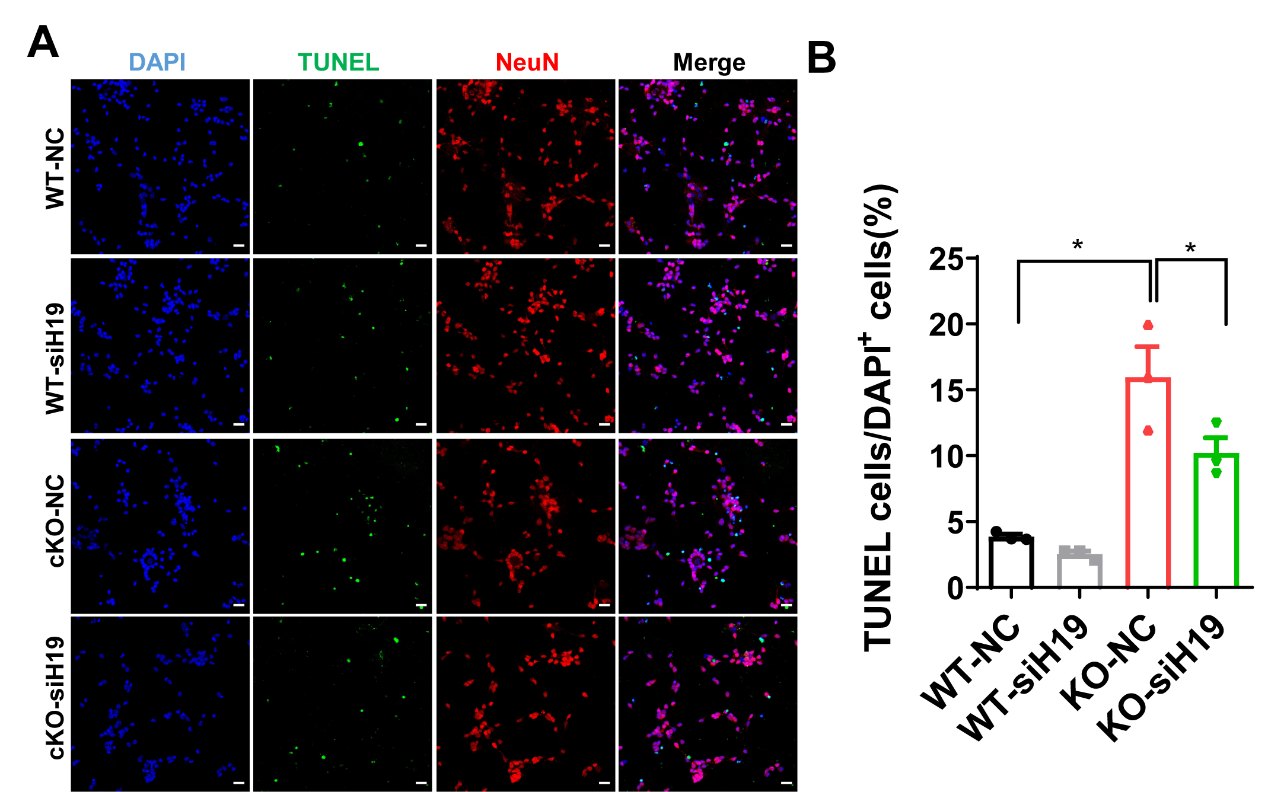
**

**Figure S7. H19 downregulation rescues cell survival in *Eed* cKO cerebellar granule cells**

(A) Immunostaining for TUNEL(green) and NeuN(red) in WT and *Eed* cKO cerebellar granule cells transfection with NC or siRNA-H19 for 48h (n > 3 experiments). Scale bars, 20 μm. (B) Quantification of TUNEL cells in WT and *Eed* cKO GCPs transfection with NC or siRNA-H19 for 48h (n > 3 experiments). **P* < 0.05, ***P*<0.01, mean ± SEM.

**
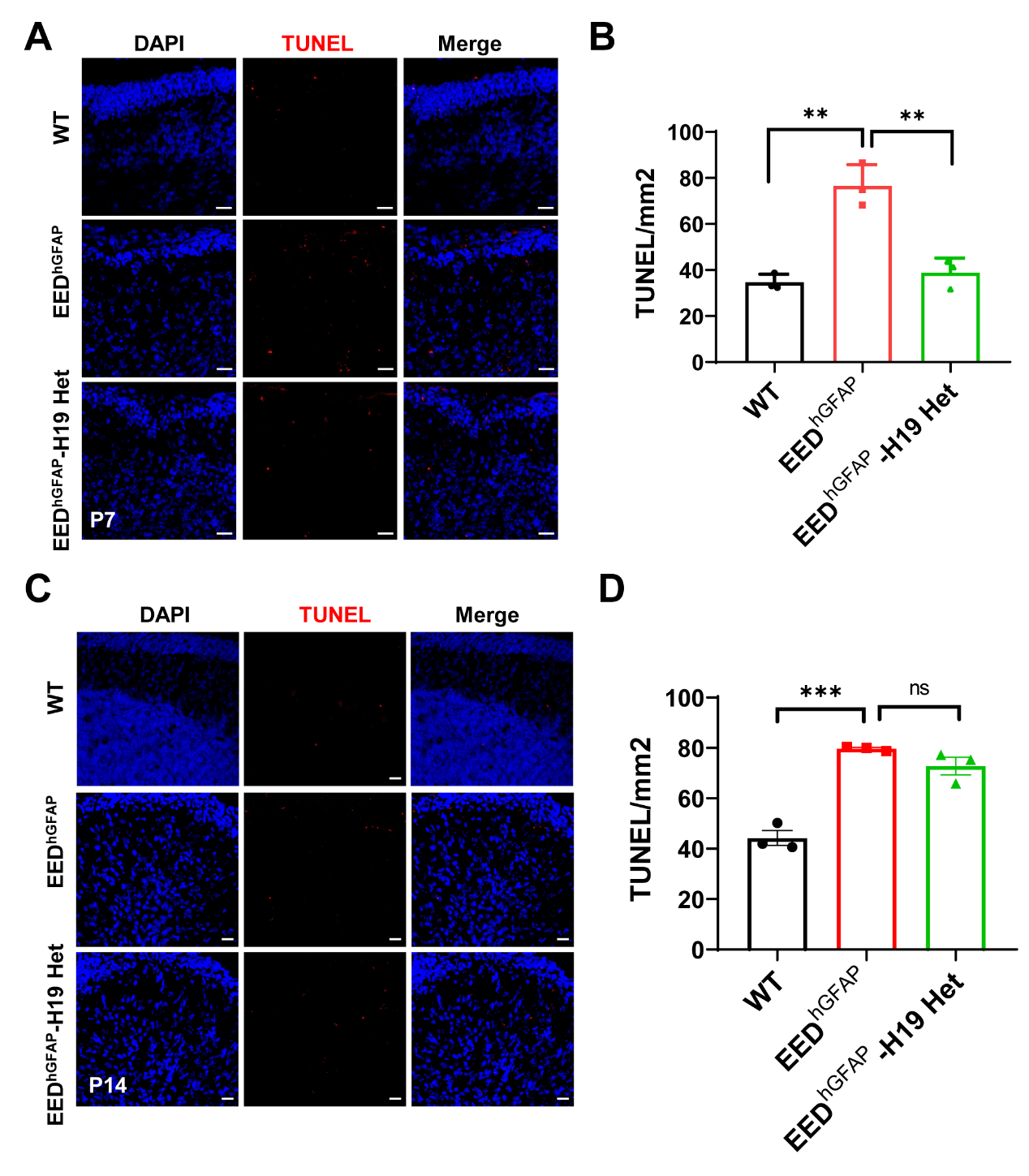
**

**Figure S8 H19 downregulation rescues cell apoptosis in *Eed* cKO mice**

(A) Immunostaining for TUNEL(red) in WT, EED^hGFAP^, and EED^hGFAP^-H19 Het mice cerebellar at P7. Scale bars, 20 μm. (B) The percentage of TUNEL-positive cells (red) per unit area was quantified. ***P* < 0.01, n = 3, mean ± SEM. (C) Immunostaining of TUNEL(red) in WT, EED^hGFAP^, and EED^hGFAP^-H19 Het mice cerebellar at P14. Scale bars, 20 μm. (D) The percentage of TUNEL-positive cells (red) per unit area was quantified. Ns, non-significant. ****P* < 0.001, n = 3, mean ± SEM.


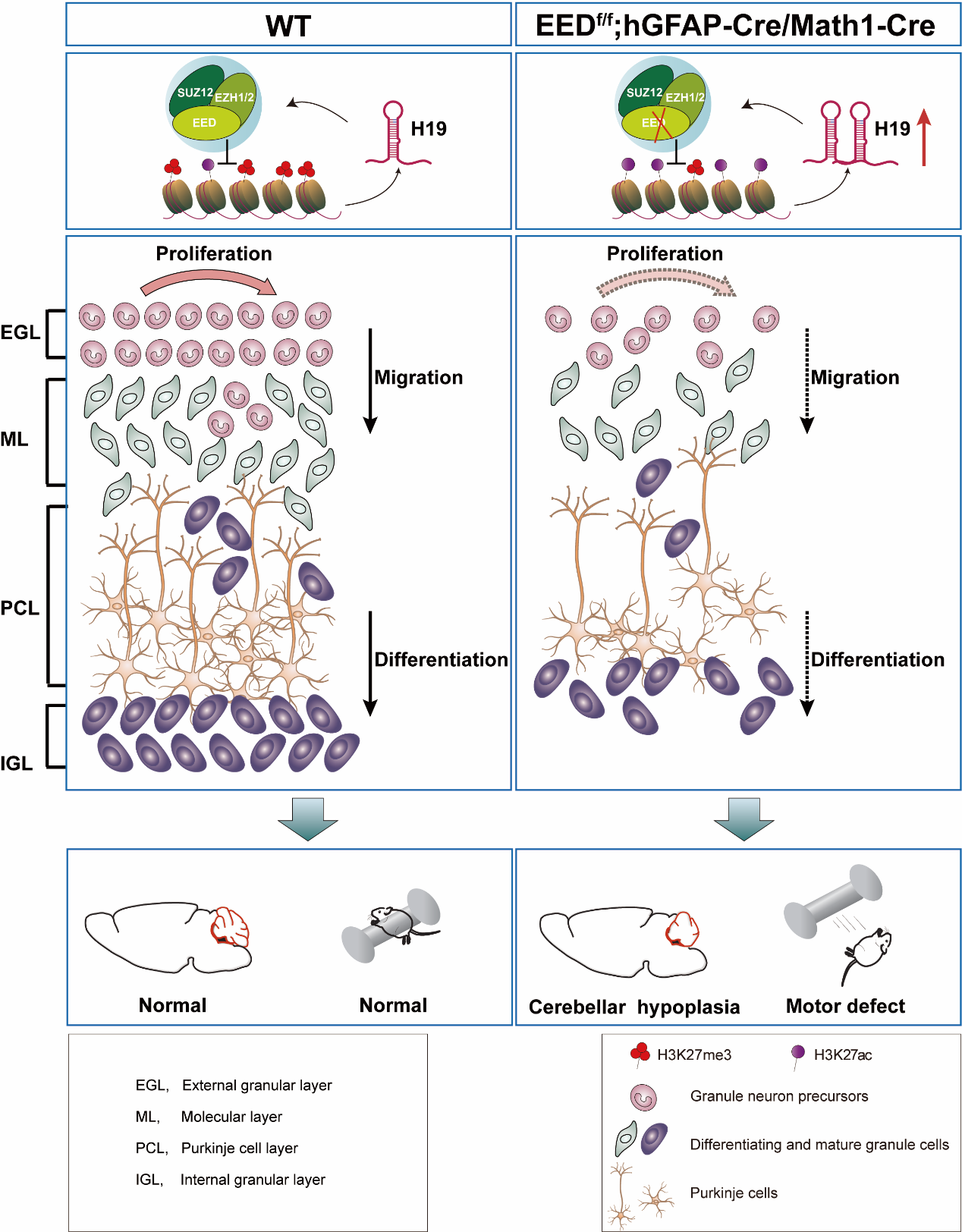


**Figure S9. A working model**

**
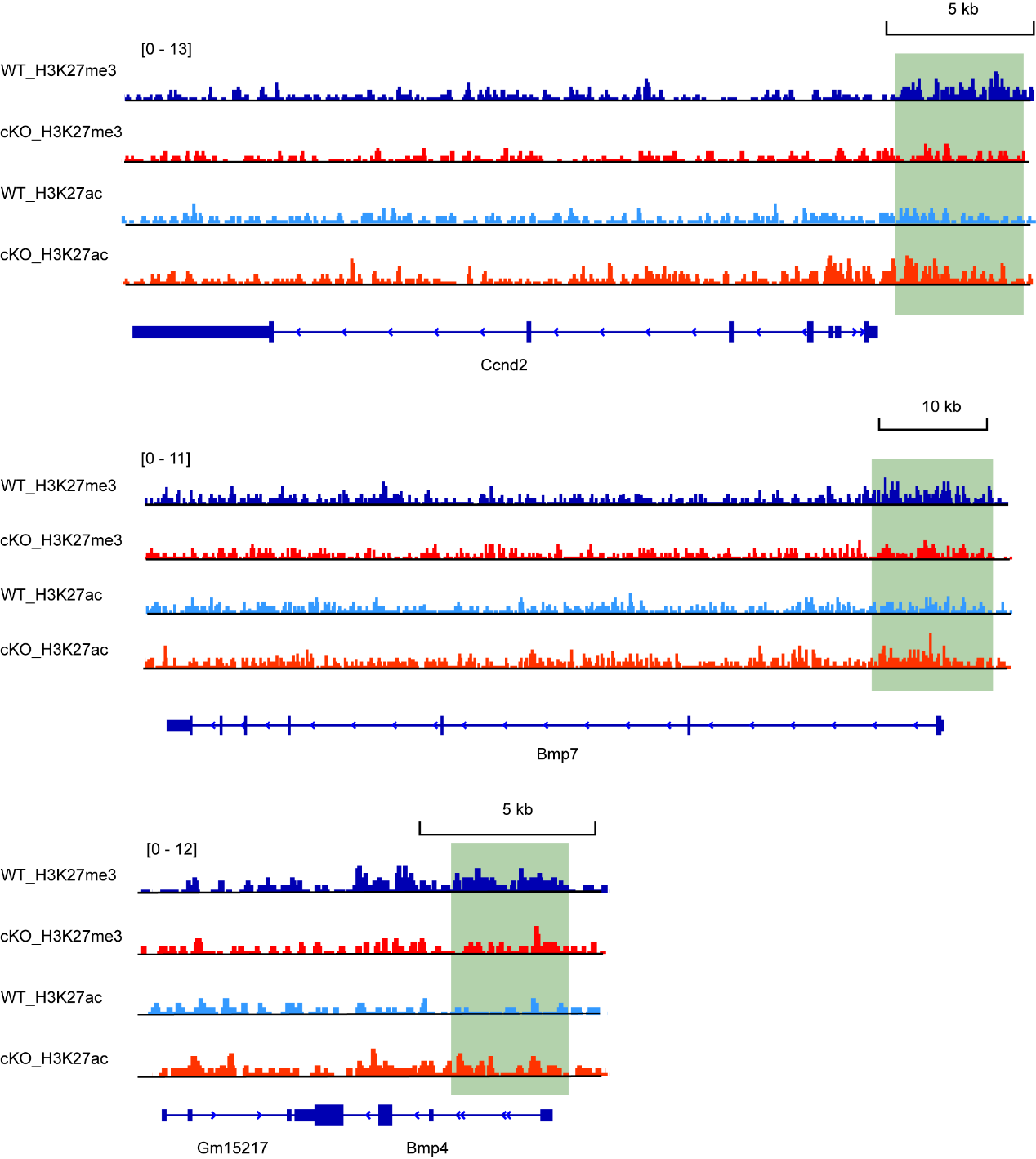
**

**Figure S10.** **The levels of the H3K27me3 and H3K27ac at the promoters of up-regulated genes found from *Eed* cKO mice**

(A) Visualization of ChIP-seq data of the H3K27me3 and H3K27ac levels of up-regulated genes (*Ccnd2*, *Bmp7*, *Bmp4*) found from *Eed* cKO mice. The regions marked in green show the gain or loss of H3K27me3 and H3K27ac.


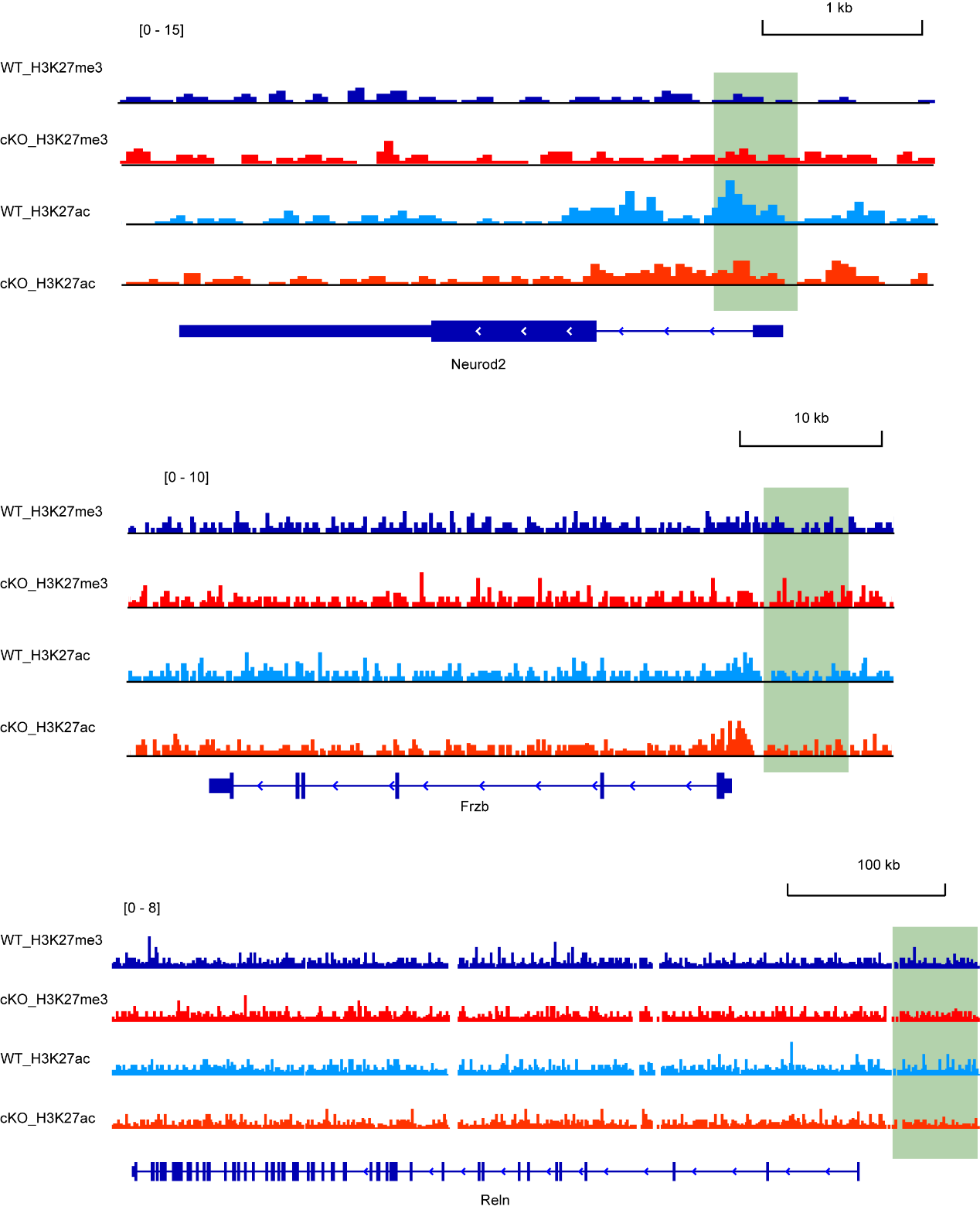


**Figure S11. The levels of the H3K27me3 and H3K27ac at the promoters of down-regulated genes found from *Eed* cKO mice**

(A) Visualization of ChIP-seq data of the H3K27me3 and H3K27ac levels of up-regulated genes (*Neurod2*, *Frzb*, *Reln*) found from *Eed* cKO mice. The regions marked in green show the gain or loss of H3K27me3 and H3K27ac.
